# *KRAS* Affects the Lipid Composition by Regulating Mitochondrial Functions and MAPK Activation in Bovine Mammary Epithelial Cells

**DOI:** 10.3390/ani12223070

**Published:** 2022-11-08

**Authors:** Wenjie Yu, Hao Jiang, Fengjiao Liu, Ze Li, Lingxia Xu, Chang Liu, Wenfa Lv, Jun Wang, Yan Gao, Shuang Liang, Nam-Hyung Kim, Jiabao Zhang, Chengzhen Chen, Bao Yuan

**Affiliations:** 1 Department of Laboratory Animals, Jilin Provincial Key Laboratory of Animal Model, Jilin University, Changchun 130062, China; 2 Department of Animal Science, Chungbuk National University, Cheongju 361-763, Korea; 3 School of Grains, Jilin Business and Technology College, Changchun 130507, China; 4 College of Animal Science and Technology, Jilin Agricultural University, Changchun 130118, China

**Keywords:** milk fat, lipid metabolism, triglyceride, *KRAS*, MAPK, endoplasmic reticulum, bovine mammary epithelial cell

## Abstract

**Simple Summary:**

Milk fat is mainly composed of triglycerides, which are synthesized in the endoplasmic reticulum of mammary epithelial cells. Many uncertain regulatory factors and mechanisms are involved in this process. In this study, we found that inhibition of the Kirsten rat sarcoma viral oncogene homolog changed the lipid composition by regulating mitochondrial functions, autophagy, endoplasmic reticulum stress, and mitogen-activated protein kinase in a dairy cow mammary epithelial cell line. These results are helpful for exploring the synthesis and secretion of milk fat at the molecular level and providing a theoretical basis for improving the flavor and nutrition of milk.

**Abstract:**

Kirsten rat sarcoma viral oncogene homolog (*KRAS*), or guanosine triphosphatase *KRAS*, is a proto-oncogene that encodes the small guanosine triphosphatase transductor protein. Previous studies have found that *KRAS* can promote cytokine secretion, cell chemotaxis, and survival. However, its effects on milk fat synthesis in bovine mammary epithelial cells are unclear. In this study, the effects of *KRAS* inhibition on cell metabolism, autophagy, oxidative stress, endoplasmic reticulum stress, mitochondrial function, and lipid composition as well as the potential mechanisms were detected in an immortalized dairy cow mammary epithelial cell line (MAC-T). The results showed that inhibition of *KRAS* changed the lipid composition (especially the triglyceride level), mitochondrial functions, autophagy, and endoplasmic reticulum stress in cells. Moreover, *KRAS* inhibition regulated the levels of the mammalian target of rapamycin and mitogen-activated protein kinase (extracellular regulated protein kinases, c-Jun N-terminal kinases, p38) activation. These results indicated that regulation of *KRAS* would affect the synthesis and composition of milk fat. These results are also helpful for exploring the synthesis and secretion of milk fat at the molecular level and provide a theoretical basis for improving the percentage of fat in milk and the yield of milk from cows.

## 1. Introduction

Among the livestock species that produce milk around the world, cows have always been the dominant species. Milk is rich in nutrition, and in addition to providing the essential nutrients for the growth and development of newborns, milk contains all the amino acids required by humans and an abundance of minerals, vitamins, milk protein, and milk fat [1]. Milk is secreted as a result of the physiological activity of the mammary glands. Mammary glands, which are vesicular skin glands and are composed mainly of mammary epithelial cells (MECs), are crucial to the reproductive process in mammals [2]. Milk production is highly dependent on the extensive development of the mammary epithelium, and this development occurs during puberty [3]. Milk fat, one of the important components of milk (approximately 4.2%), is mainly composed of triglycerides (TGs, approximately 98%), which are synthesized in the endoplasmic reticulum (ER) of MECs [4]. High-yielding dairy cows tend to have a higher risk of ketosis [5]. The etiology of ketosis is the establishment of a state of negative energy balance (NEB) around calving. It is a process that happens when a cow does not have enough carbohydrates to burn for energy. Mammary epithelial cells of high-yielding dairy cows often require more energy supply due to their higher milk production, thereby leading to oxidative stress and ER stress [6]. Therefore, studies on the processes, factors, and mechanisms involved in milk fat and milk protein production in MECs are very important for improving the quality and yield of milk.

The synthesis of milk fat by bovine MECs (BMECs) is associated with many important processes, such as fatty acid (FA) transport, de novo FA synthesis, FA channeling and metabolism, and lipid droplet formation [7]. CD36 is the most important protein that regulates the transport of long-chain FAs into mammary cells [8]. Most long-chain FAs that are imported into cells are activated by acyl-CoA synthetase long-chain family member 1 (ACSL1) to form long-chain acetyl-CoA [9]. After long-chain FA or long-chain acetyl-CoA binds to fatty acid-binding protein (FABP) 3, the molecules can directly participate in TG formation with the help of glycerol-3-phosphate acyltransferase (GPAM) or be used as a substrate by stearoyl-CoA desaturase (SCD) to indirectly synthesize TG with the help of FABP4 [10]. De novo FA synthesis is carried out by acetyl-CoA carboxylase (ACC) and fatty acid synthase (FASN) utilizing acetyl-CoA and butyryl-CoA [11]. The formation of acetyl-CoA from acetate is performed by acyl-CoA synthetase short-chain family member 2 (ACSS2) [12].

After lipid droplets are formed, the milk fat globule membrane (MFGM) can stabilize the structure of the droplet [13]. Phospholipids make up approximately 30% of the total lipid content of MFGM, and the three prominent lipids are sphingomyelin (SM), phosphatidylcholine (PC), and phosphatidylethanolamine (PE), which account for 85% of the total phospholipids [14]. Other important polar lipids present in the membrane include glycerophospholipids (GPs), phosphatidylserine (PS), phosphatidylinositol (PI), and gangliosides (GGs) [15]. The peroxisome proliferator-activated receptor gamma (PPARG) expression level is significantly upregulated and participates in milk fat metabolism by PPARG coactivator 1 alpha and lipin 1 (LPIN1) during lactation [16]. PPARG also affects sterol regulatory element-binding protein (SREBP) activity by regulating insulin-induced gene 1 expression. Activated SREBP1 translocates to the nucleus and participates in the regulation of lipid-related gene expression [17]. Moreover, when the synthesis and milk fat secretion demands are high, the number of mitochondria significantly increases to provide sufficient ATP in MECs during lactation and meet the increasing energy requirements [18]. Because the ER has a high level of synthesis and secretion and a remarkable cell metabolism, BMECs generally suffer when the ER is under stress and oxidative stress. In general, stress can be relieved by the redox system, but the continuous production of milk fat and an overly abundant milk yield seriously harm mammary health and even lead to ketosis [19].

As an oncogene, Kirsten rat sarcoma viral oncogene homolog (*KRAS*) has previously been found to promote cell survival, cytokine secretion, and chemotaxis. *KRAS* is also an important factor and therapeutic target of cancer because it can mediate glutamine metabolism and regulate the nuclear factor E2-related factor 2, ral guanine nucleotide dissociation stimulator, TENSIN 4, and phosphatidylinositol 3-kinase (PI3K) pathways [20,21]. Previous studies indicated that inhibition of *KRAS* affected lipid accumulation, which is a key step in milk fat synthesis. The expression level of *KRAS* significantly decreased with adipogenic differentiation, which was associated with changes in *KRAS* expression during preadipocyte differentiation [22,23]. Moreover, *KRAS*, a member of the RAS family, can affect adipogenesis by regulating mitogen-activated protein kinase (MAPK) in various cell types [24,25]. The MAPK signaling pathway is a key pathway in milk fat synthesis and the entire lactation process [26,27]. Therefore, we predicted that *KRAS* also affects milk fat synthesis in cows.

MAC-T, the immortalized bovine mammary epithelial cell line, is a cell model for researching mammary developmental biology, mammary pathology, mammary bioreactors, and lactation engineering [28,29,30]. It can also be used to explore the effects of functional genes [31,32] and compounds [33] on the development of mammary components and milk fat synthesis. In this study, we investigated the effects of *KRAS* inhibition on autophagy, mitochondrial functions, oxidative stress, ER stress, cell metabolites, lipid composition, and MAPK activation in MAC-T cells. This study is helpful for understanding the synthesis and secretion of milk fat and provides a theoretical basis for improving the percentage of fat in milk and the yield of milk from cows. At the same time, the results may also provide new directions for an in-depth understanding of the prevention, occurrence, and therapy of ketosis in dairy cows.

## 2. Materials and Methods

All reagents and chemicals were purchased from Sigma-Aldrich (St. Louis, MO, USA), unless indicated otherwise.

### 2.1. Cell Culture

MAC-T cells (Qingqi Biotechnology Development Co., Ltd., Shanghai, China) were cultured with Dulbecco’s Modified Eagle Medium with Ham’s F12 (DMEM/F12) medium (HyClone, South Logan, UT, USA) containing 10% fetal bovine serum (Gibco, Grand Island, NE, USA) at 37 °C in a 5% CO_2_ atmosphere.

### 2.2. Cell Transfection

In brief, MAC-T cells were plated in 12-well plates (NEST Biotechnology, Wuxi, China). Then, the culture medium was replaced with DMEM/F12. Lipofectamine 2000 Transfection Reagent (Invitrogen, Rochester, NY, USA)/siRNA (GenePharma, Suzhou, China) complexes were added at 1:2 ratios to the plates. The final concentration of siRNAs was 80 nM in DMEM/F12 medium. After 6 h, the medium was changed to DMEM/F12 with 10% fetal bovine serum, and the culture plates were incubated for 18 h. The siRNA sequences are shown in Supplementary Table S1.

### 2.3. Autophagy Inhibition by Chloroquine

In brief, MAC-T cells were cultured in 12-well plates. Then, the cells were treated with 50 μM chloroquine (Selleck, dissolved in DMSO) for 24 h when they reached 70% confluence. Next, the autophagy levels were detected by Western blot and immunofluorescence assays.

### 2.4. Apoptosis Detection

Cell apoptosis was detected with flow cytometry (Beckman Coulter, Brea, CA, USA) according to the instructions provided in Annexin V-FITC apoptosis analysis kit (Tianjin Sungene Biotech Co., Ltd., Tianjin, China). The details of the materials and methods are shown in the Section S1.

### 2.5. RNA Extraction and Quantitative Real-Time Reverse Transcription Polymerase Chain Reaction (qRT-PCR)

In brief, total RNA of MAC-T cells was extracted by using TRIzol Reagent (Life Technologies, Carlsbad, CA, USA). Gene expression was quantified by the Mastercycler ep realplex (Eppendorf, Hamburg, Germany) and 2^−△△CT^ method with *β-ACTIN* as the standard. The details of the methods and the primer sequences are shown in the Section S1 and Table S2.

### 2.6. Protein Separation and Western Blot Analysis

In brief, MAC-T cells were trypsinized and collected. Then, RIPA buffer (Solarbio, Beijing, China) containing 1% PMSF (100 mM, Solarbio) was used for lysis. The GAPDH loading controls were performed on the same membrane unless indicated otherwise. The details of the methods and antibodies used in the study are shown in the Section S1 and Table S3.

### 2.7. Immunofluorescence

In brief, a total of 1 × 10^5^ cells were seeded on 12-well plates containing cell culture slides (NEST Biotechnology). Twenty-four hours after transfection, we performed immunofluorescence detection. The details of the methods and antibodies used in the study are shown in the Section S1 and Table S3.

### 2.8. TG Assay

In brief, intracellular TG was detected using a tissue/cell triacylglycerol assay kit (Applygen Technologies, Beijing, China). The details of the materials and methods are shown in the Section S1.

### 2.9. Reactive Oxygen Species (ROS) Assay

In brief, 24 h after cell transfection, intracellular ROS levels were detected using 10 µM 2′,7′-dichlorofluorescein diacetate (DCFH-DA, Beyotime, Shanghai, China) and flow cytometry (Beckman Coulter, Brea, CA, USA). The details of the materials and methods are shown in the Section S1.

### 2.10. Total Antioxidant Capacity (T-AOC), Catalase (CAT), and Glutathione (GSH) Assays

According to the manufacturer’s requirements, 24 h after cell transfection, a T-AOC assay kit (Solarbio), CAT assay kit (Solarbio), and reduced GSH assay kit (Solarbio) were used to detect the levels of T-AOC, CAT, and GSH, respectively. The absorption value was measured with a microplate reader (Tecan, Mannedorf, Switzerland). The T-AOC and activity of GSH and CAT were calculated based on the absorption value and standard curve.

### 2.11. Mitochondrial Morphology Assay

Briefly, mitochondrial morphology was detected using 200 nM MitoTracker Red CMXRos (Invitrogen). The details of the materials and methods are shown in the Section S1.

### 2.12. ATP Assay

Briefly, intracellular ATP levels were measured using an enhancing ATP detection kit (Beyotime). The details of the materials and methods are shown in the Section S1.

### 2.13. Mitochondrial DNA (mtDNA) Copy Number Assay

The whole genome was extracted by using the QIAamp Fast DNA Tissue Kit (Qiagen, Hilden, Germany). mtDNA was detected using qPCR. The details of the materials and methods are shown in the Section S1.

### 2.14. Metabolomics Analysis

In brief, 24 h after transfection, MAC-T cells were washed with PBS and digested by trypsin for collection. After sample extraction, the samples were injected into chromatography and mass spectrometry systems for detection. Then, the raw data were processed by using Progenesis QI (Waters Corporation, Milford, MA, USA). For the data extracted by the Metabolites Search, metabolites with RSD > 30% were deleted. Significantly differentially expressed metabolites between the si-NC and si-*KRAS* groups were identified with cutoff values of *p*-value < 0.05, log_2_(FC) > 1, and VIP > 1. The details of the materials and methods are shown in the Section S1.

### 2.15. Lipidomics Analysis

In brief, 24 h after transfection, MAC-T cells were washed with PBS and digested by trypsin for collection. After sample extraction, the samples were injected into chromatography and mass spectrometry systems for detection. Then, the raw data were processed by using LipidSearch (Thermo Fisher, Waltham, MA, USA). For data extracted by Lipid Search, lipid molecules with RSD > 30% were deleted. Significantly differentially expressed lipids between the si-NC and si-*KRAS* groups were identified with cutoff values of *p*-value < 0.05, log_2_(FC) > 1, and VIP > 1. The details of the materials and methods are shown in the Section S1.

### 2.16. Statistical Analysis

All experiments were performed three times unless indicated otherwise. Data are represented as the means ± standard deviation. A Bonferroni post hoc test was used to compare the data. All statistical analyses were performed using the SPSS software (version 21.0, IBM, Chicago, IL, USA). *p*-Values < 0.05 were considered statistically significant.

## 3. Results

### 3.1. KRAS Inhibition Mainly Affects Lipid Metabolites in MAC-T Cells

**Figure 1:**
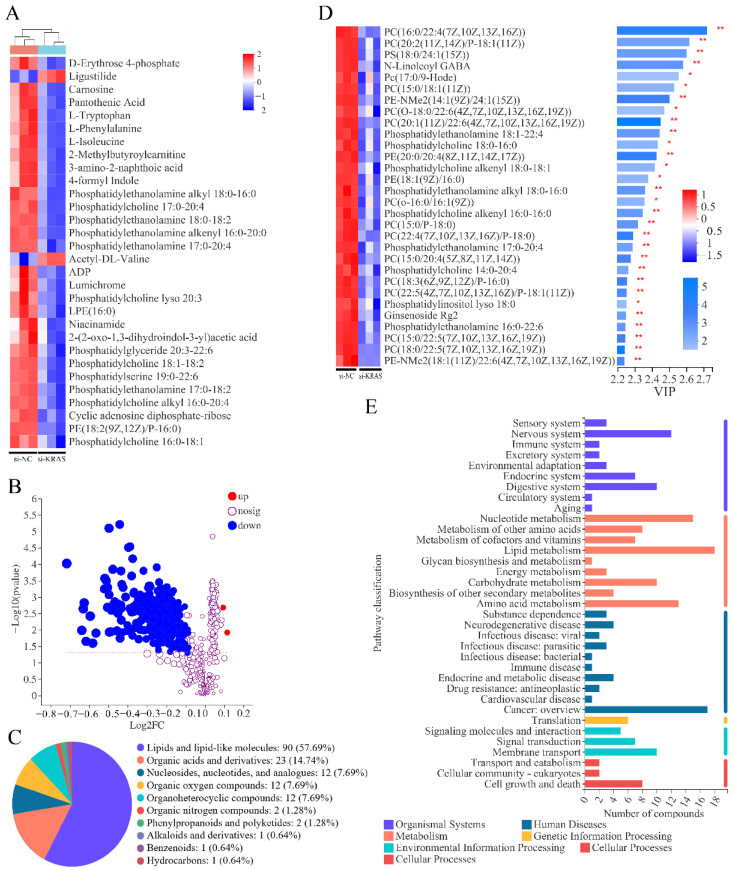
Effects of Kirsten rat sarcoma viral oncogene homolog (*KRAS*) on cell metabolism in MAC-T cells. (**A**) Heatmap of some of the differential metabolites. In the figure, each column represents a sample, each row represents a metabolite, and the color indicates the relative level of metabolites expressed in the group. Red indicates that the metabolite is expressed at high levels, and blue indicates lower expression. (**B**) Volcano plot of the differentially expressed metabolites. Each dot in the figure represents a metabolite, and the size of the dot indicates the variable importance in the projection (VIP) value. The blue dots are significantly downregulated metabolites, and the red dots are significantly upregulated metabolites. (**C**) Pie chart based on HMDB chemical taxonomy (Super Class) counts for the differential metabolites identified. The color indicates different classes, and the area size indicates the number of metabolites. (**D**) Top 30 differential metabolites from the VIP plot based on the OPLS-DA models. (**E**) KEGG pathway enrichment of differential metabolites. Significant differences are represented with * (*p* < 0.05) and ** (*p* < 0.01).

The immunofluorescence results showed that *KRAS* was localized in the nuclei of MAC-T cells (Supplementary Figure S1). Western blot results indicated that *KRAS*-specific siRNA (si-*KRAS*) significantly inhibited the protein expression level of *KRAS* in MAC-T cells (Supplementary Figure S1). After determining the effectiveness of siRNA, we conducted metabolomics analysis. The results showed that the levels of 291 known metabolites were significantly changed with *KRAS* inhibition (Figure 1B); only 2 metabolites were significantly upregulated, and the rest were significantly downregulated. Based on the classification of HMDB, 57% of 156 (matched in the HMDB 4.0 database) differential metabolites were lipid metabolites, followed by 14.74% organic acids and derivatives (Figure 1C). Moreover, to further analyze feature metabolites, a variable importance in the projection (VIP) plot from the OPLS was carried out to select distinct variables as potential metabolites for exploring the effects of *KRAS* on cell metabolism in MAC-T cells. We found that there were 28 lipid metabolites in the top 30 metabolites with VIP scores (Figure 1D). KEGG enrichment analysis showed that the differential metabolites were mainly enriched in the lipid metabolism pathway (Figure 1E). The detailed data are shown in Supplementary Tables S4 and S5.

### 3.2. KRAS Inhibition Affects the Lipid Composition in MAC-T Cells

**Figure 2:**
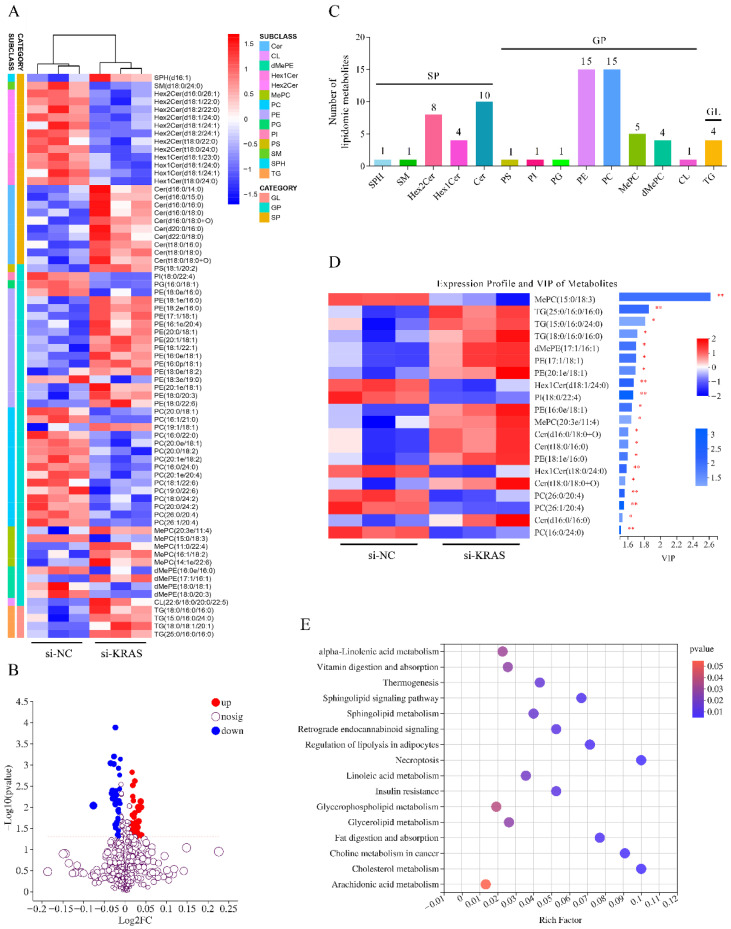
Effects of Kirsten rat sarcoma viral oncogene homolog (*KRAS*) on lipid composition in MAC-T cells. (**A**) Heatmap of all the differential lipidomic metabolites after *KRAS* inhibition. In the figure, each column represents a sample, each row represents a metabolite, and the color indicates the relative level of metabolites expressed in the group. Red indicates that the metabolite is expressed at high levels, and blue indicates lower expression. (**B**) Volcano plot of the differentially lipidomic metabolites. Each dot in the figure represents a metabolite, and the size of the dot indicates the variable importance in the projection (VIP) value. The blue dots are significantly downregulated metabolites, and the red dots are significantly upregulated metabolites. (**C**) Identified differential lipidomic metabolites were classified. (**D**) Differential metabolites with VIP > 1.5 from the VIP plot based on the OPLS-DA models. (**E**) KEGG pathway enrichment of differential lipidomic metabolites. The size of bubbles in the figure represents the amount of differential metabolites, and the color of bubbles represents the *p*-values. Significant differences are represented with * (*p* < 0.05) and ** (*p* < 0.01).

The above results indicate that *KRAS* inhibition mainly affects lipid metabolism in MAC-T cells. To further explore the effects of *KRAS* on lipid composition, we carried out a lipidomic study. As shown in Figure 2A,B, after *KRAS* inhibition, 71 lipid metabolites were differentially expressed, and 24 kinds of sphingolipids (SPs), 43 kinds of GPs, and 4 kinds of glycerides (GLs) were found (Figure 2C). All the differentially expressed GLs were TG, including TG (18:0/16:0/16:0), TG (15:0/16:0/24:0), TG (18:0/18:1/20:1), and TG (25:0/16:0/16:0). PS, cardiolipin (CL), and most PE and methylphosphocholine (MePC) were significantly increased, while phosphatidylglycerol (PG), PI, and most PC and dimethyl phosphatidyl ethanolamine (dMePE) were decreased in the differentially expressed GP. Among the differentially expressed SPs, sphingosine (SPH) and ceramide (Cer) were significantly increased, while SM, hexosylceramide (Hex1Cer), and dihexosylceramide (Hex2Cer) were significantly decreased (Figure 2A,C). To select potential lipidomic biomarkers, VIP scores were used to rank the contribution of metabolites to the discrimination between the si-*KRAS* group and si-negative control (NC) groups based on the weighted coefficients of the OPLS-DA model. The results showed that the metabolite with the highest VIP score was MePC (15:0/18:3, VIP = 2.62), followed by TG (25:0/16:0/16:0, VIP = 1.85), TG (15:0/16:0/24:0, VIP = 1.81), and TG (18:0/16:0/16:0, VIP = 1.71, Figure 2D). The KEGG pathway enrichment results indicated that these 71 differential metabolites were mainly enriched in the sphingolipid metabolism, glycerophospholipid metabolism, fat digestion, and absorption pathways (Figure 2E). Detailed data are shown in Supplementary Table S6.

### 3.3. KRAS Inhibition Improved Milk Fat Synthesis in MAC-T Cells

**Figure 3:**
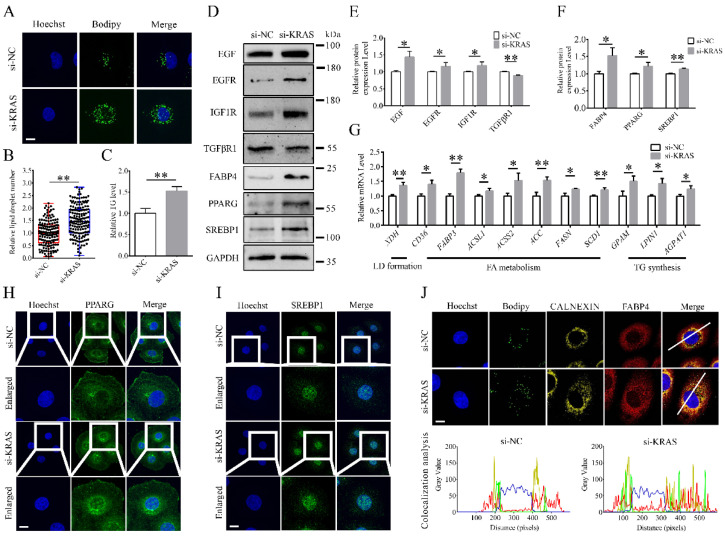
Effects of Kirsten rat sarcoma viral oncogene homolog (*KRAS*) on the synthesis of milk fat in MAC-T cells. (**A**,**B**) Representative Bodipy staining images and relative changes in milk fat content with or without *KRAS* inhibition. Bar = 10 μm. The experiments were performed five times. The red line indicates the si-NC group, the blue line indicates the si-*KRAS* group. (**C**) Relative triglyceride (TG) level changes in MAC-T cells with or without *KRAS* inhibition. (**D**–**F**) Representative Western blot images of peroxisome proliferator-activated receptor gamma (PPARG), sterol regulatory element-binding protein (SREBP1), fatty acid-binding protein (FABP) 4, epidermal growth factor (EGF), EGF receptor (EGFR), IGF1 receptor (IGF1R), and transforming growth factor beta receptor type 1 (TGFβR1) and the relative protein level assays in MAC-T cells with or without *KRAS* inhibition. (**G**) Relative mRNA expression levels of xanthine dehydrogenase (*XDH*), *CD36*, *FABP3*, acyl-CoA synthetase long-chain family member 1 (*ACSL1*), acyl-CoA synthetase short-chain family member 2 (*ACSS2*), acetyl-CoA carboxylase (*ACC*), fatty acid synthase (*FASN*), stearoyl-CoA desaturase 1 (*SCD1*), glycerol-3-phosphate acyltransferase (*GPAM*), lipin 1 (*LPIN1*), and 1-acylglycerol-3-phosphate O-acyltransferase 1 (*AGPAT1*) with or without *KRAS* inhibition in MAC-T cells. (**H**,**I**) Representative immunofluorescence images of PPARG and SREBP1 in MAC-T cells with or without *KRAS* inhibition. Bar = 10 μm. (**J**) Representative immunofluorescence images of Bodipy, FABP4, and CALNEXIN colocalization analysis in MAC-T cells with or without *KRAS* inhibition. Bar = 10 μm. The fluorescence signals of Hoechst, Bodipy, CALNEXIN, and FABP4 were represented by the blue, green, yellow, and red lines, respectively. Fluorescence colocalization was analyzed based on the white line in merged image. Significant differences are represented with * (*p* < 0.05) and ** (*p* < 0.01).

After *KRAS* inhibition, intracellular lipid droplet content and TG content were significantly increased in MAC-T cells (Figure 3A–C). The protein levels of FABP4, PPARG, SREBP1, epidermal growth factor (EGF), EGF receptor (EGFR), and IGF1 receptor (IGF1R) were significantly increased with *KRAS* inhibition (Figure 3D–F), while the protein level of transforming growth factor beta receptor type 1 (TGFβR1) was significantly decreased with *KRAS* inhibition in MAC-T cells. In addition, after *KRAS* inhibition, the mRNA levels of xanthine dehydrogenase (*XDH*), *CD36*, *FABP3*, *ACSL1*, *ACSS2*, *ACC*, *FASN*, *SCD1*, *GPAM*, *LPIN1*, and 1-acylglycerol-3-phosphate O-acyltransferase 1 (*AGPAT1*) were also significantly increased (Figure 3G). In addition, the immunofluorescence results showed that the nuclear expression levels of PPARG (Figure 3H) and SREBP1 (Figure 3I) in MAC-T cells were significantly increased, and the colocalization level of lipid droplets and FABP4 with ER was significantly enhanced with *KRAS* inhibition (Figure 3J). Detailed data are shown in Supplementary Table S4.

### 3.4. KRAS Inhibition Improved Mitochondrial Function

**Figure 4:**
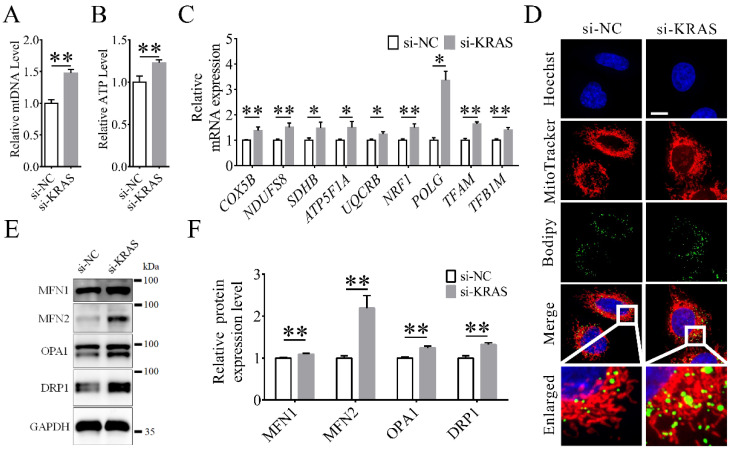
Effects of Kirsten rat sarcoma viral oncogene homolog (*KRAS*) on mitochondrial function in MAC-T cells. (**A**) Relative level of mitochondrial DNA (mtDNA) copy number in MAC-T cells with or without *KRAS* inhibition. (**B**) Relative ATP level in MAC-T cells with or without *KRAS* inhibition. (**C**) Relative mRNA expression levels of cytochrome c oxidase subunit 5B (*COX5B*), NADH:ubiquinone oxidoreductase core subunit S8 (*NDUFS8*), succinate dehydrogenase complex iron sulfur subunit B (*SDHB*), ATP synthase F1 subunit alpha (*ATP5F1A*), ubiquinol–cytochrome c reductase binding protein (*UQCRB*), nuclear respiratory factor 1 (*NRF1*), DNA polymerase gamma catalytic subunit (*POLG*), mitochondrial transcription factor A (*TFAM*), and mitochondrial transcription factor B1 (*TFB1M*) in MAC-T cells with or without *KRAS* inhibition. (**D**) Representative Hoechst, MitoTracker, and Bodipy fluorescence images in MAC-T cells with or without *KRAS* inhibition. Bar = 10 μm. (**E**,**F**) Representative Western blot images and relative protein expression levels of mitofusin (MFN)1, MFN2, optic atrophy 1 (OPA1), and dynamin-related protein 1 (DRP1) in MAC-T cells with or without *KRAS* inhibition. Significant differences are represented with * (*p* < 0.05) and ** (*p* < 0.01).

The results showed that the levels of mtDNA (Figure 4A) and ATP (Figure 4B) significantly increased with *KRAS* inhibition in MAC-T cells. The mRNA levels of the mitochondrial-related genes cytochrome c oxidase subunit 5B (*COX5B*), NADH:ubiquinone oxidoreductase core subunit S8 (*NDUFS8*), succinate dehydrogenase complex iron sulfur subunit B (*SDHB*), ATP synthase F1 subunit alpha (*ATP5F1A*), and ubiquinol–cytochrome c reductase binding protein (*UQCRB*) and the mitochondrial biogenesis-related genes nuclear respiratory factor 1 (*NRF1*), DNA polymerase gamma catalytic subunit (*POLG*), mitochondrial transcription factor A (*TFAM*), and mitochondrial transcription factor B1 (*TFB1M*) in the *KRAS* inhibition group were also significantly increased (Figure 4C). Interestingly, with MitoTracker staining, we found mitochondrial morphological changes in some of the MAC-T cells that produced more lipids (Figure 4D). Moreover, the expression levels of the mitochondrial fusion-related proteins mitofusin (MFN)1, MFN2, and optic atrophy 1 (OPA1) and fission-related protein dynamin-related protein 1 (DRP1) were significantly increased with *KRAS* inhibition (Figure 4E,F). Detailed data are shown in Supplementary Table S4.

### 3.5. KRAS Inhibition Enhanced ER Stress in MAC-T Cells

**Figure 5:**
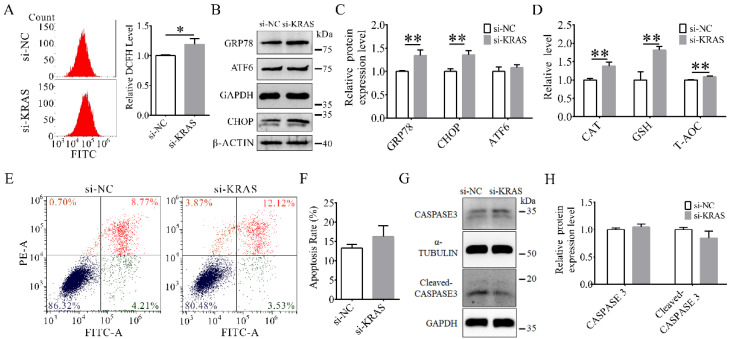
Effects of Kirsten rat sarcoma viral oncogene homolog (*KRAS*) on reactive oxygen species (ROS) and endoplasmic reticulum (ER) stress in MAC-T cells. (**A**) Representative flow cytometry images of ROS content and relative dichlorofluorescein (DCFH) levels in MAC-T cells with or without *KRAS* inhibition. (**B**,**C**) Representative Western blot images and relative protein levels of glucose-regulated protein 78 (GRP78), activating transcription factor 6 (ATF6), and C/EBP homologous protein (CHOP) in MAC-T cells with or without *KRAS* inhibition. The β-ACTIN loading controls were performed for CHOP on the same membrane. (**D**) Relative levels of catalase (CAT), glutathione (GSH), and total antioxidant capacity (T-AOC) in MAC-T cells with or without *KRAS* inhibition. (**E**,**F**) Representative flow cytometry images and apoptosis rate in MAC-T cells with or without *KRAS* inhibition. (**G**,**H**) Representative Western blot images and relative protein expression levels of CASPASE 3 and cleaved-CASPASE 3 in MAC-T cells with or without *KRAS* inhibition. Significant differences are represented with * (*p* < 0.05) and ** (*p* < 0.01).

The flow cytometry results indicated that the ROS level was significantly increased in the *KRAS* inhibition group in MAC-T cells (Figure 5A). The protein levels of glucose-regulated protein (GRP) 78 and C/EBP homologous protein (CHOP) were also significantly increased, while the level of ATF6 was not significantly changed with *KRAS* inhibition (Figure 5B,C). In addition, the relative levels of CAT, GSH, and T-AOC were significantly increased (Figure 5D). The apoptosis levels and protein expression levels of CASPASE 3 and cleaved-CASPASE 3 were not significantly changed (Figure 5E–H). Detailed data are shown in Supplementary Table S4.

### 3.6. KRAS Inhibition Enhanced Autophagy in MAC-T Cells

**Figure 6:**
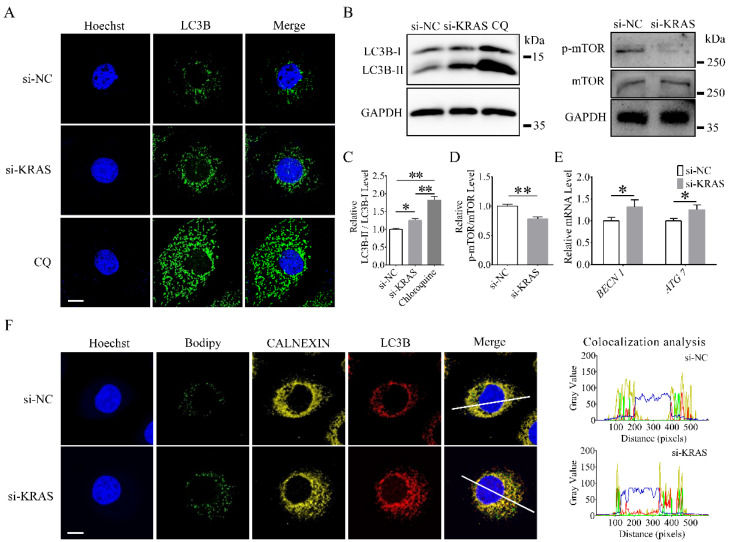
Effects of Kirsten rat sarcoma viral oncogene homolog (*KRAS*) on autophagy in MAC-T cells. (**A**) Representative immunofluorescence images of microtubule-associated protein 1 light chain 3 beta (LC3B) in MAC-T cells with or without *KRAS* inhibition. Bar = 10 μm. (**B**–**D**) Representative Western blot images and relative protein levels of LC3B, mammalian target of rapamycin (mTOR), and p-mTOR in MAC-T. (**E**) Relative mRNA levels of beclin 1 (*BECN1*) and autophagy-related 7 (*ATG7*) in MAC-T cells with or without *KRAS* inhibition. (**F**) Representative immunofluorescence images and colocalization analysis of Bodipy, LC3B, and CALNEXIN in MAC-T cells with or without *KRAS* inhibition. Bar = 10 μm. The fluorescence signals of Hoechst, Bodipy, CALNEXIN, and LC3B were represented by the blue, green, yellow, and red lines, respectively. Fluorescence colocalization was analyzed based on the white line in merged image. Significant differences are represented with * (*p* < 0.05) and ** (*p* < 0.01).

Among MAC-T cells, the immunofluorescence results indicated that the numbers of microtubule-associated protein 1 light chain 3 beta (LC3B) puncta in the cytoplasm were significantly increased in the *KRAS* inhibition and chloroquine-treated groups compared with the NC group (Figure 6A), and the protein levels of LC3B-II/LC3B-I in the *KRAS* inhibition and chloroquine-treated groups were also increased (Figure 6B,C). The relative levels of phosphorylated mTOR versus total mTOR (Figure 6B,D) were significantly decreased, while the mRNA levels of beclin 1 (*BECN1*) and autophagy-related 7 (*ATG7*) were significantly increased after *KRAS* inhibition (Figure 6E). In addition, after *KRAS* inhibition, the colocalization level of CALNEXIN, LC3B, and Bodipy was enhanced, as shown by an immunofluorescence assay (Figure 6F). Detailed data are shown in Supplementary Tables S4 and S7.

### 3.7. KRAS Inhibition Regulated MAPK Activation

**Figure 7:**
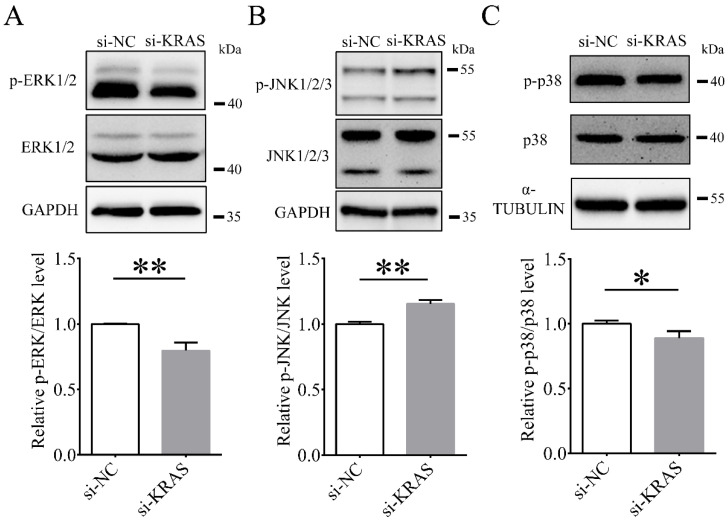
Effects of Kirsten rat sarcoma viral oncogene homolog (*KRAS*) on the phosphorylation levels of mitogen-activated protein kinases (MAPKs) in MAC-T cells. Representative Western blotting images and phosphorylated/total levels of extracellular regulated protein kinase 1/2 (ERK1/2, (**A**)), c-Jun N-terminal kinase 1/2/3 (JNK1/2/3, (**B**)), and p38 (**C**) in MAC-T cells with or without *KRAS* inhibition. The α-TUBULIN loading controls were performed for p-p38 and p38 on the same membrane. Significant differences are represented with * (*p* < 0.05) and ** (*p* < 0.01).

Western blotting was used to detect the MAPKs (extracellular regulated protein kinases, ERKs; c-Jun N-terminal kinases, JNKs; p38) phosphorylation levels in MAC-T cells with or without *KRAS* treatment. As shown in Figure 7, compared with the si-NC group, the si-*KRAS*-treated group exhibited significantly increased phosphorylation levels of ERK1/2, JNK1/2/3, and p38. Detailed data are shown in Supplementary Table S4.

## 4. Discussion

Milk fat is one of the main nutrients in milk. It not only can provide energy for newborn calves but is also an important raw material for many dairy products [34]. Milk fat content is closely related to the quality and flavor of milk and even affects the development of animal husbandry. Milk fat, which is composed mainly of TG, is synthesized by the ER of BMECs [35]. During lactation, mammary mitochondria and ER have been suggested to play important roles in milk lipid biosynthesis. When excessive lipid synthesis and secretion occurs, the mitochondria and ER will continue to experience stress and damage. To maintain homeostasis, cells try to increase autophagy to remove damaged ER and excess lipids. Previous studies have shown that *KRAS* affects lipid accumulation. However, the specific roles of *KRAS* in the lactation process remain unclear.

### 4.1. KRAS Affects Lipid Accumulation

In mammalian mammary glands, most luminal epithelial cells express cytokeratin 18 (CK18) [36]. Through immunofluorescence studies (Supplementary Figure S1), we found that CK18 was strongly expressed in all MAC-T cells, which indicated that MAC-T cells were purified and had the characteristic of BMECs. *KRAS* inhibition leads to increased expression levels of PPARG and SREBP1, suggesting that inhibition of *KRAS* may affect the localization of key nuclear transcription factors, thus improving the ability to promote lipid droplet formation in MAC-T cells [37]. Increased levels of *CD36*, FABP4, *ACSL1*, *ACC*, *FASN*, *SCD1*, and *AGPAT6* [38] also indicated that *KRAS* inhibition may increase lipogenic gene expression; the transcriptional activation of these genes is consistent with the abundant milk fat synthesis that occurs at the onset and throughout lactation. Moreover, genes related to FA metabolism, TG synthesis, and lipid droplet formation, such as *ACSS2*, *GPAM*, *LPIN1*, and *XDH*, were also increased after *KRAS* inhibition. Once FAs with >10 carbons are formed, they are activated by ACSL1 and bound to FABP3, which allows the FAs to enter the TG synthesis pathway [39]. Short-chain FAs are inserted into the TG via diacylglycerol O-acyltransferase 1 [40]. Once TG is formed, it is inserted into the intraleaflet of the ER membrane to form lipid droplets. Perilipin 2 is essential for the formation of lipid droplets [41] and for the secretory pathway involving butyrophilin subfamily 1 member A1, and XDH also seems to play a role in the mechanism that involves perilipin 2 and butyrophilin subfamily 1 member A1 [42]. These results indicated that nuclear *KRAS* may mediate signaling that can activate or deactivate some transcription factors and affect the translocation of PPARG, SREBP1, and FABP4 to regulate TG formation in MAC-T cells. Moreover, previous studies have also shown that IGF-1 promotes adipogenesis [43], while blocking IGF-1 signaling disrupts adipogenesis [44]. However, TGFβ and TGFβR1 exhibited opposite effects on those of IGF1 on lipid formation. TGFβ inhibits adipocyte differentiation but is expressed by adipocytes [45]. TGFβ receptors strongly decrease as adipogenesis proceeds [46]. In this study, *KRAS* inhibition significantly increased the protein expression levels of IGF1R and decreased the protein expression levels of TGFβR1 in MAC-T cells, which suggests that *KRAS* has potential roles in regulating the IGF and TGF signaling pathways. The increased levels of EGF and EGFR expression after *KRAS* inhibition also indicate that *KRAS* can regulate the process of intracellular lipid formation and lipid accumulation [47].

### 4.2. Inhibition of KRAS Caused Cell Stress

The ER is the only place where milk fat synthesis occurs, and its activity changes during lactation [48,49]. During lactation, the ER activity of BMECs is greatly increased and even stressed because a high level of milk fat synthesis and secretion is necessary and its cellular metabolism is substantial. Changes in the ER stress pathway at different stages in the lactation cycle are normal adaptations of the tissues to the changing physiological state [50]. GRP78, or BiP, is a central regulator of ER stress due to its role as a major ER chaperone, its antiapoptotic properties, and its ability to control the activation of transmembrane ER stress sensors (inositol-requiring enzyme 1, RNA-dependent protein kinase-like ER kinase, and ATF6) through a binding–release mechanism [51]. ER stress mediates SREBP1/2 activation as well as the accumulation of TG and cholesterol [52]. Previous studies indicated that compared with the perinatal stage (day 15), the expression levels of RNA-dependent protein kinase-like ER kinase, ATF3, CHOP, and GRP78 significantly increased in the mammary gland of a cow during lactation, and the expression levels of ATF6, X-box binding protein 1, GRP94, and membrane bound transcription factor peptidase site 1 also showed the same trend [50]. These results further demonstrate that the *KRAS* inhibition-mediated ER stress-signaling pathway may be associated with lipogenesis. Curiously, although increased milk fat formation often leads to increased ER stress, the protein levels of GRP78 and CHOP were significantly improved, while ATF6 levels were not changed significantly after *KRAS* inhibition. These results showed that among MAC-T cells, the regulation of *KRAS* in ER stress may be different from the result that the expression of GRP78 can be activated by ATF6, a target of the ER stress response, which has been implicated in the ER stress response pathway [53]. This may mean that an increased level of milk fat synthesis does not always lead to increased levels of ER stress, in other words, ER stress from a high production level of milk fat and protein secretion may be reduced or blocked by other pathways. In addition, studies have shown that ER stress can lead to apoptosis [53]. Our results showed that the levels of CASPASE 3, cleaved-CASPASE 3 [54], and apoptosis did not change significantly with *KRAS* inhibition. These results indicated that *KRAS*-mediated ER stress did not induce cell apoptosis in MAC-T cells. However, this may be affected by the knockdown efficiency of siRNA.

Moreover, the MECs of lactating cows undergo intensive metabolism in cells and accumulate a large amount of free radicals, such as ROS [55]. MECs in cows that yield high levels of milk are more likely to produce ROS due to their increased capacity to synthesize and secrete milk [55]. However, excessive ROS accumulation incapacitates the redox system, which leads to oxidative stress and cell damage [56]. However, our study showed that although the level of ROS increased with *KRAS* inhibition, the total capacity to produce milk fat and mitochondrial function were not negatively affected in MAC-T cells. This indicated that after *KRAS* inhibition, despite the increase in ROS under the intensity metabolic influence, oxidative homeostasis was maintained, as the antioxidant defenses were sufficient. After *KRAS* inhibition, oxidative homeostasis may be maintained by the significantly increased levels of GSH and CAT.

### 4.3. Inhibition of KRAS Enhanced Mitochondrial Function

Mitochondria are recognized as essential components for meeting energy needs and maintaining metabolic homeostasis because mtDNA is a template for several polypeptides in respiratory chain complexes that are essential for ATP generation [57]. The decrease in mammary mitochondrial ATP synthesis on day 21 postpartum correlates with previously reported decreases in milk production in mice [58]. These results are consistent with the idea that ATP synthesis activity in mitochondria is temporally correlated with the production of milk during lactation. Combined with our results, *KRAS* may affect the synthesis of milk fat by regulating the level of mtDNA, intracellular ATP, and the related gene expression of the mitochondrial electron transport chain (*NDUFS8*, *SDHB*, *UQCRB*, *COX5B*, and *ATP5F1A*) and mitochondrial biogenesis (*NRF1*, *TFAM*, *TFB1M*, and *POLG*) [59]. Interestingly, after *KRAS* inhibition, some MAC-T cells with higher lipid production contained mitochondria with morphological changes. Through electron microscopy, previous studies found that during the early postpartum period, both the size and density of secretory cell mitochondria increase, which also indicated that the increase in milk production during early lactation is linked with a dramatic increase in mammary mitochondrial number [49]. However, it is difficult to determine whether the mitochondrial morphological changes that occur with *KRAS* inhibition lead to the increased level of their own biosynthesis and ATP [60] or whether the changes are caused by ROS or ER stress only based on the changes in mitochondrial morphology [61]. Therefore, we further examined fusion- and fission-related proteins. There is a close connection between mitochondrial dynamics and biosynthesis [62]. Studies have shown that the better the mitochondrial quality states are, the stronger the fusion–fission and biogenesis cycles [63]. The mitochondrial function and biosynthesis capacity of cells were significantly increased, and the highly expressed mitochondrial dynamics-related genes OPA1, MFN2, FIS1, and DRP1 were enhanced by leptin treatment [64]. This is precisely related to the inhibition of mitochondrial activity and biosynthesis by rotenone treatment, accompanied by the downregulation of DRP1, OPA1, and MFN2 protein expression levels [65]. Our results showed that the expression levels of the mitochondrial fusion-related proteins MFN1, MFN2, and OPA1 and the fission-related protein DRP1 were significantly increased after *KRAS* inhibition. These results suggested that after *KRAS* inhibition, the highly dynamic mitochondrial fusion and fission cycle was proposed to balance the compensation of damage by fusion and the elimination of damage by fission, eventually leading to an increase in mitochondrial biosynthesis.

### 4.4. Inhibition of KRAS Induced Cell Autophagy

After *KRAS* inhibition, the number of autophagosomes, protein levels of LC3B-II/LC3B-I, and mRNA levels of *BECN1* [66] and *ATG7* [67] were significantly increased, while the phosphorylation level of mTOR [68] was significantly decreased in MAC-T cells.

Chloroquine inhibits the degradation of autophagosomes, thereby inhibiting autophagy levels in cells. During this process, the mRNA expression levels of *ATG7* and *BECN 1* will not increase. However, in this study, *KRAS* inhibition resulted in increased mRNA levels of *ATG7* and *BECN 1*. These results indicated that the inhibition of *KRAS* indeed increases the level of autophagy rather than preventing the degradation of autophagosomes and inhibiting autophagy in MAC-T cells. In addition, the colocalization level of autophagosomes with ER and lipid droplets was enhanced after *KRAS* inhibition, which indicates that the level of selective autophagy, that is, ER-phagy and lipophagy, was enhanced. ER-phagy and lipophagy represent the major quality control mechanisms that ensure that exhausted or harmful ERs are properly renewed and that the balance of lipid metabolism is maintained [69]. This indicated that after *KRAS* inhibition, TG levels were significantly increased, and lipid metabolism was disordered, so lipophagy may be initiated to maintain lipid homeostasis. When excessive lipid synthesis and secretion occurs, the ER suffers from stress and damage, and the level of intracellular ROS also increases significantly, which may together lead to an increased level of ER-phagy. Many studies have shown that the activation or inhibition of mTOR can upregulate or downregulate milk fat levels, respectively [70,71]. Interestingly, the finding that the phosphorylation level of mTOR decreased and milk fat levels increased with *KRAS* inhibition was the opposite of what we observed in our research. This may be because after *KRAS* inhibition, the increased level of stress caused by excessive TG accumulation inhibits mTOR activation, which enhances autophagy levels to maintain the homeostasis of intracellular environments [68,72].

### 4.5. KRAS Affects MAPK Activation

ERK, p38, and JNK MAPKs are intracellular signaling pathways that play a pivotal role in adipogenesis [73]. In this study, *KRAS* inhibition decreased the levels of ERK activation and PPARG expression, which suggests that after *KRAS* inhibition, the levels of FAS expression and lipid accumulation were increased by enhancing the expression of C/EBPα/β and PPARG that was caused by ERK inhibition [74]. Conversely, the activation of the ERK pathway inhibited lipid droplet formation, and the protein expression levels of the lipid-related genes C/EBPα, PPARG, lipoprotein lipase, and FABP4 were associated with our hypothesis [75]. Some studies have shown that the inhibition of p38 activity can promote lipid formation [76]. In this study, *KRAS* inhibition also decreased the phosphorylation level of p38, indicating that *KRAS* has the potential to regulate the activation of the nuclear factor of activated T cells 4 (NFATc4) and enhance the nuclear translocation of NFATc4 after dephosphorylation by NFAT, thus promoting the expression of PPARc and lipid formation [77]. The inhibition of p38 by microtubule affinity regulating kinase 4 also promoted adipogenesis, which together indicates that the activation of ERK and p38 can inhibit the capacity to adipogenesis. JNK activity also plays important roles in adipogenesis. Previous studies have shown that microtubule affinity regulating kinase 4 can also promote lipid formation by inhibiting p38 and activating JNK1, and JNK activation promotes mesenchymal stem cell adipogenic differentiation [78]; these results seem to suggest that JNK plays opposite roles to ERK and p38 in adipogenesis. In this study, the differential expression of related genes also demonstrated that *KRAS* inhibition can activate the JNK pathway, thus promoting lipid formation by regulating PPARG and FABP4 [79]. After *KRAS* inhibition, the phosphorylation level of JNK increased, while the levels of ERK and p38 decreased at the same time, which suggests that *KRAS* plays important roles in lipid accumulation in MAC-T cells.

### 4.6. KRAS Regulates Milk Fat Composition

The metabolomics analysis showed that after *KRAS* inhibition, the intracellular metabolite levels changed significantly; most of these metabolites were lipid metabolites, and the top 30 metabolites with VIP scores also contained 28 lipid metabolites. As the main component of milk fat, TG also regulates cellular homeostasis and the levels of other lipid metabolites [80]. Previous studies have shown that TG has negative regulatory effects with lysophosphatidylinositol/PI [81]. This is consistent with our results that after *KRAS* inhibition, various forms of TG were significantly increased, and one form of PI was significantly decreased. Moreover, the levels of SM, Hex1Cer, and Hex2Cer were negatively correlated with the Cer and SPH levels after *KRAS* inhibition. The Cer formed from SM turnover might be hydrolyzed by ceramidases to SPH [82,83]. Cellular accumulations of Cer and SPH, which occur in response to stress, such exposure to tumor necrosis factor alpha or oxidative stress, are associated with apoptotic responses [84]. SP not only plays important roles in cell signal transduction but also acts as an important part of MFGM together with GP [85]. After *KRAS* inhibition, various forms of SP and GP were differentially expressed, and VIP score analysis also showed that TG was the main contributor to the difference between groups. These results indicated that *KRAS* inhibition has important effects on both the milk fat globule core and MFGM and that the cellular stress caused by excessive TG accumulation promotes the transformation of Hex1Cer, Hex2Cer, and SM into Cer and SPH. This result also seems to indicate that *KRAS*-mediated TG level changes in the milk fat globule core may lead to the different MFGM components.

**Figure 8:**
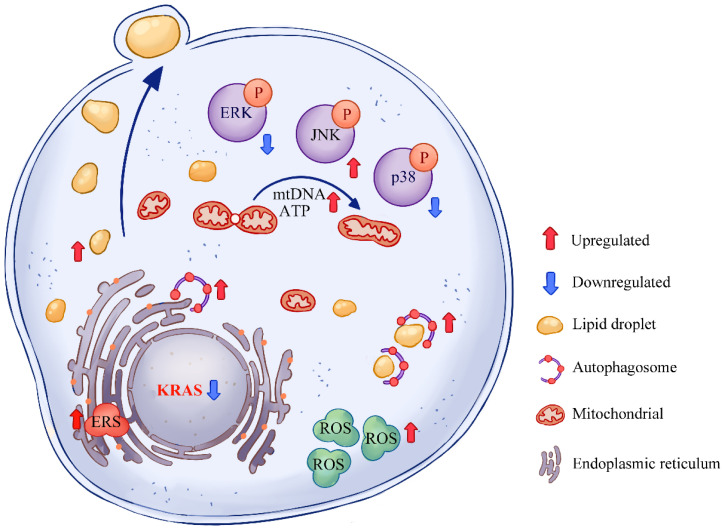
Schematic diagram of the effects of Kirsten rat sarcoma viral oncogene homolog (*KRAS*) inhibition in MAC-T cells. After *KRAS* inhibition, the levels of triglycerides, mitochondrial DNA (mtDNA), ATP, reactive oxygen species (ROS), autophagy, and endoplasmic reticulum stress (ERS) were upregulated in MAC-T cells. Moreover, the levels of mitogen-activated protein kinase (extracellular regulated protein kinases, ERK; c-Jun N-terminal kinases, JNK; p38) activation were regulated after *KRAS* inhibition in MAC-T cells.

Overall, the levels of TG, mitochondrial functions, ROS, ERS, and autophagy were increased in MAC-T cells with *KRAS* inhibition. The phosphorylated levels of MAPKs (ERK, JNK, and p38) were also changed after *KRAS* inhibition (Figure 8). Moreover, after *KRAS* inhibition, the levels of some lipids were also regulated in addition to TG, such as PC, PE, Cer, and HexCer. These findings broaden the understanding of the effects of *KRAS* on milk fat metabolism and provide a new theoretical basis for further exploring the mechanism of milk fat synthesis and secretion. With *KRAS* inhibition, the mitochondrial function and milk fat synthesis capacity of MAC-T cells were significantly enhanced, while intracellular ROS and ER stress levels were increased. Thus, although *KRAS* inhibition enhanced milk fat secretion, it simultaneously stressed mammary epithelial cells and enhanced autophagy levels, which is not good for the health of the cows. Conversely, relatively high *KRAS* expression may lead to a decrease in milk fat secretion, which is detrimental to offspring feeding. Therefore, although *KRAS* is a key regulator of milk fat secretion, simply changing its expression level up- or downregulation may be detrimental to the health of either the next generation or dairy cows. This is similar to the occurrence of ketosis in mammary tissue, which suggests that abnormal expression of *KRAS* may cause a series of symptoms by affecting energy imbalance and play important roles in ketosis. Therefore, the regulation of *KRAS* may also provide new directions for exploring the specific mechanism and treatment of ketosis.

## 5. Conclusions

In MAC-T cells, *KRAS* regulated the phosphorylation levels of ERK, p38, and JNK. After *KRAS* inhibition, mitochondrial functions were improved. However, ROS and ERS were also increased. This would also lead to an increase in intracellular autophagy to maintain oxidative homeostasis and remove damaged ER. Excessive TG accumulation caused by *KRAS* inhibition led to intensive lipid metabolism and secretion, which suggests that *KRAS* plays important roles in lipid accumulation.

## Data Availability

The data presented in this study are available in the article or Supplementary Material.

## Supplementary Materials

The following are available online at https://www.mdpi.com/article/10.3390/ani12223070/s1. Section S1: Supplementary Materials and Methods; Figure S1: *KRAS* is expressed mainly in MAC-T cell nuclei. Table S1: siRNA sequences used in this study; Table S2: Primer sequences used in this study; Table S3: Antibodies used for Western Blot and immunofluorescence (IF); Table S4: All values of folder changes (si-*KRAS* vs. si-NC); Table S5: Differential metabolites from metabolomics analysis with *KRAS* inhibition; Table S6: Differential lipid metabolites from lipidomics analysis with *KRAS* inhibition; Table S7: Relative folder changes of LC3B-II/LC3B-I.

## References

[1] Willett W.C., Ludwig D.S. (2020). Milk and health. N. Engl. J. Med..

[2] Martignani E., Accornero P., Miretti S., Baratta M. (2018). Bovine Mammary Organoids: A Model to Study Epithelial Mammary Cells. Methods Mol. Biol..

[3] Finot L., Chanat E., Dessauge F. (2018). Molecular signature of the putative stem/progenitor cells committed to the development of the bovine mammary gland at puberty. Sci. Rep..

[4] Sharmin M.M., Mizusawa M., Hayashi S., Arai W., Sakata S., Yonekura S. (2020). Effects of fatty acids on inducing endoplasmic reticulum stress in bovine mammary epithelial cells. J. Dairy Sci..

[5] Shin E.K., Jeong J.K., Choi I.S., Kang H.G., Hur T.Y., Jung Y.H., Kim I.H. (2015). Relationships among ketosis, serum metabolites, body condition, and reproductive outcomes in dairy cows. Theriogenology.

[6] Hassan F.U., Nadeem A., Javed M., Saif-Ur-Rehman M., Shahzad M.A., Azhar J., Shokrollahi B. (2022). Nutrigenomic Interventions to Address Metabolic Stress and Related Disorders in Transition Cows. Biomed. Res. Int..

[7] Hanuš O., Samková E., Křížová L., Hasoňová L., Kala R. (2018). Role of Fatty Acids in Milk Fat and the Influence of Selected Factors on Their Variability-A Review. Molecules.

[8] Daquinag A.C., Gao Z., Fussell C., Immaraj L., Pasqualini R., Arap W., Akimzhanov A.M., Febbraio M., Kolonin M.G. (2021). Fatty acid mobilization from adipose tissue is mediated by CD36 posttranslational modifications and intracellular trafficking. JCI Insight.

[9] Li T., Li X., Meng H., Chen L., Meng F. (2020). ACSL1 affects Triglyceride Levels through the PPARγ Pathway. Int. J. Med. Sci..

[10] Zhou F., Xue J., Shan X., Qiu L., Miao Y. (2022). Functional roles for AGPAT6 in milk fat synthesis of buffalo mammary epithelial cells. Anim. Biotechnol..

[11] Wallace M., Metallo C.M. (2020). Tracing insights into de novo lipogenesis in liver and adipose tissues. Semin. Cell Dev. Biol..

[12] Xu H., Luo J., Ma G., Zhang X., Yao D., Li M., Loor J.J. (2018). Acyl-CoA synthetase short-chain family member 2 (ACSS2) is regulated by SREBP-1 and plays a role in fatty acid synthesis in caprine mammary epithelial cells. J. Cell Physiol..

[13] Brink L.R., Lönnerdal B. (2020). Milk fat globule membrane: The role of its various components in infant health and development. J. Nutr. Biochem..

[14] Manoni M., Di Lorenzo C., Ottoboni M., Tretola M., Pinotti L. (2020). Comparative Proteomics of Milk Fat Globule Membrane (MFGM) Proteome across Species and Lactation Stages and the Potentials of MFGM Fractions in Infant Formula Preparation. Foods.

[15] Murthy A.V.R., Guyomarc’h F., Lopez C. (2016). The temperature-dependent physical state of polar lipids and their miscibility impact the topography and mechanical properties of bilayer models of the milk fat globule membrane. Biochim. Biophys. Acta.

[16] Bionaz M., Loor J.J. (2008). Gene networks driving bovine milk fat synthesis during the lactation cycle. BMC Genom..

[17] Lee J.E., Schmidt H., Lai B., Ge K. (2019). Transcriptional and Epigenomic Regulation of Adipogenesis. Mol. Cell. Biol..

[18] Favorit V., Hood W.R., Kavazis A.N., Skibiel A.L. (2021). Graduate Student Literature Review: Mitochondrial adaptations across lactation and their molecular regulation in dairy cattle. J. Dairy Sci..

[19] Song Y., Loor J.J., Li C., Liang Y., Li N., Shu X., Yang Y., Feng X., Du X., Wang Z. (2021). Enhanced mitochondrial dysfunction and oxidative stress in the mammary gland of cows with clinical ketosis. J. Dairy Sci..

[20] Liu P., Wang Y., Li X. (2019). Targeting the untargetable *KRAS* in cancer therapy. Acta Pharm. Sin. B.

[21] Zhang Y., Liu J.L., Wang J. (2020). *KRAS* gene silencing inhibits the activation of PI3K-Akt-mTOR signaling pathway to regulate breast cancer cell epithelial-mesenchymal transition, proliferation and apoptosis. Eur. Rev. Med. Pharmacol. Sci..

[22] Yu W., Chen C.Z., Peng Y., Li Z., Gao Y., Liang S., Yuan B., Kim N.H., Jiang H., Zhang J.B. (2021). *KRAS* Affects Adipogenic Differentiation by Regulating Autophagy and MAPK Activation in 3T3-L1 and C2C12 Cells. Int. J. Mol. Sci..

[23] Elsafadi M., Manikandan M., Almalki S., Mobarak M., Atteya M., Iqbal Z., Hashmi J.A., Shaheen S., Alajez N., Alfayez M. (2018). TGFβ1-Induced Differentiation of Human Bone Marrow-Derived MSCs Is Mediated by Changes to the Actin Cytoskeleton. Stem. Cells Int..

[24] Ryan S.L., Matheson E., Grossmann V., Sinclair P., Bashton M., Schwab C., Towers W., Partington M., Elliott A., Minto L. (2016). The role of the RAS pathway in iAMP21-ALL. Leukemia.

[25] Tajan M., Paccoud R., Branka S., Edouard T., Yart A. (2018). The RASopathy Family: Consequences of Germline Activation of the RAS/MAPK Pathway. Endocr. Rev..

[26] Cecchinato A., Macciotta N.P.P., Mele M., Tagliapietra F., Schiavon S., Bittante G., Pegolo S. (2019). Genetic and genomic analyses of latent variables related to the milk fatty acid profile, milk composition, and udder health in dairy cattle. J. Dairy Sci..

[27] Bhat S.A., Ahmad S.M., Ibeagha-Awemu E.M., Bhat B.A., Dar M.A., Mumtaz P.T., Shah R.A., Ganai N.A. (2019). Comparative transcriptome analysis of mammary epithelial cells at different stages of lactation reveals wide differences in gene expression and pathways regulating milk synthesis between Jersey and Kashmiri cattle. PLoS ONE.

[28] Saeed S.I., Aklilu E., Mohammedsalih K.M., Adekola A.A., Mergani A.E., Mohamad M., Kamaruzzaman N.F. (2021). Antibacterial Activity of Ikarugamycin against Intracellular Staphylococcus aureus in Bovine Mammary Epithelial Cells In Vitro Infection Model. Biology.

[29] Mitz C.A., Viloria-Petit A.M. (2019). TGF-beta signalling in bovine mammary gland involution and a comparative assessment of MAC-T and BME-UV1 cells as in vitro models for its study. PeerJ.

[30] Liu X., Shen J., Zong J., Liu J., Jin Y. (2021). Beta-Sitosterol Promotes Milk Protein and Fat Syntheses-Related Genes in Bovine Mammary Epithelial Cells. Animals.

[31] Han L.Q., Gao T.Y., Yang G.Y., Loor J.J. (2018). Overexpression of SREBF chaperone (SCAP) enhances nuclear SREBP1 translocation to upregulate fatty acid synthase (FASN) gene expression in bovine mammary epithelial cells. J. Dairy Sci..

[32] Su B.Q., Han Y.Q., Fan S.S., Ming S.L., Wan B., Lu W.F., Chu B.B., Yang G.Y., Wang J. (2018). PKM2 knockdown influences SREBP activation and lipid synthesis in bovine mammary-gland epithelial MAC-T cells. Biotechnol. Lett..

[33] Zhong W., Shen J., Liao X., Liu X., Zhang J., Zhou C., Jin Y. (2020). Camellia (*Camellia oleifera Abel*.) seed oil promotes milk fat and protein synthesis-related gene expression in bovine mammary epithelial cells. Food Sci. Nutr..

[34] Viriato R.L.S., Queirós M.S., Macedo G.A., Ribeiro A.P.B., Gigante M.L. (2022). Design of new lipids from bovine milk fat for baby nutrition. Crit. Rev. Food Sci. Nutr..

[35] Cheng J., Zhang Y., Ge Y., Li W., Cao Y., Qu Y., Liu S., Guo Y., Fu S., Liu J. (2020). Sodium butyrate promotes milk fat synthesis in bovine mammary epithelial cells via GPR41 and its downstream signalling pathways. Life Sci..

[36] Park H.J., Lee W.Y., Jeong H.Y., Song H. (2016). Regeneration of Bovine Mammary Gland in Immunodeficient Mice by Transplantation of Bovine Mammary Epithelial Cells Mixed with Matrigel. Int. J. Stem. Cells.

[37] Zhang M.Q., Gao J.L., Liao X.D., Huang T.H., Zhang M.N., Wang M.Q., Tian Y., Bai J., Zhou C.H. (2019). miR-454 regulates triglyceride synthesis in bovine mammary epithelial cells by targeting PPAR-γ. Gene.

[38] Tian H., Luo J., Shi H., Chen X., Wu J., Liang Y., Li C., Loor J.J. (2020). Role of peroxisome proliferator-activated receptor-α on the synthesis of monounsaturated fatty acids in goat mammary epithelial cells. J. Anim. Sci..

[39] Tian Z., Zhang Y., Zhang H., Sun Y., Mao Y., Yang Z., Li M. (2022). Transcriptional regulation of milk fat synthesis in dairy cattle. J. Funct. Foods.

[40] Sun Y., Luo J., Zhu J., Shi H., Li J., Qiu S., Wang P., Loor J.J. (2016). Effect of short-chain fatty acids on triacylglycerol accumulation, lipid droplet formation and lipogenic gene expression in goat mammary epithelial cells. Anim. Sci. J..

[41] Cruz A.L.S., Barreto E.A., Fazolini N.P.B., Viola J.P.B., Bozza P.T. (2020). Lipid droplets: Platforms with multiple functions in cancer hallmarks. Cell Death Dis..

[42] Han L., Zhang M., Xing Z., Coleman D.N., Liang Y., Loor J.J., Yang G. (2020). Knockout of butyrophilin subfamily 1 member A1 (BTN1A1) alters lipid droplet formation and phospholipid composition in bovine mammary epithelial cells. J. Anim. Sci. Biotechnol..

[43] Wang F., Li H., Lou Y., Xie J., Cao D., Huang X. (2019). Insulin-like growth factor I promotes adipogenesis in hemangioma stem cells from infantile hemangiomas. Mol. Med. Rep..

[44] Ambele M.A., Dhanraj P., Giles R., Pepper M.S. (2020). Adipogenesis: A Complex Interplay of Multiple Molecular Determinants and Pathways. Int. J. Mol. Sci..

[45] Choy L., Derynck R. (2003). Transforming growth factor-beta inhibits adipocyte differentiation by Smad3 interacting with CCAAT/enhancer-binding protein (C/EBP) and repressing C/EBP transactivation function. J. Biol. Chem..

[46] Zhang Z., Gao Y., Xu M.Q., Wang C.J., Fu X.H., Liu J.B., Han D.X., Jiang H., Yuan B., Zhang J.B. (2019). miR-181a regulate porcine preadipocyte differentiation by targeting TGFBR1. Gene.

[47] Cao S., Pan Y., Tang J., Terker A.S., Arroyo Ornelas J.P., Jin G.N., Wang Y., Niu A., Fan X., Wang S. (2022). EGFR-mediated activation of adipose tissue macrophages promotes obesity and insulin resistance. Nat. Commun..

[48] Wu C.C., Howell K.E., Neville M.C., Yates J.R., McManaman J.L. (2000). Proteomics reveal a link between the endoplasmic reticulum and lipid secretory mechanisms in mammary epithelial cells. Electrophoresis.

[49] Dai W., White R., Liu J., Liu H. (2022). Organelles coordinate milk production and secretion during lactation: Insights into mammary pathologies. Prog. Lipid Res..

[50] Invernizzi G., Naeem A., Loor J.J. (2012). Short communication: Endoplasmic reticulum stress gene network expression in bovine mammary tissue during the lactation cycle. J. Dairy Sci..

[51] Kumar V., Maity S. (2021). ER Stress-Sensor Proteins and ER-Mitochondrial Crosstalk-Signaling Beyond (ER) Stress Response. Biomolecules.

[52] Kim J.Y., Garcia-Carbonell R., Yamachika S., Zhao P., Dhar D., Loomba R., Kaufman R.J., Saltiel A.R., Karin M. (2018). ER Stress Drives Lipogenesis and Steatohepatitis via Caspase-2 Activation of S1P. Cell.

[53] Zhang Z., Zhang L., Zhou L., Lei Y., Zhang Y., Huang C. (2019). Redox signaling and unfolded protein response coordinate cell fate decisions under ER stress. Redox Biol..

[54] Green D.R. (2022). Caspase Activation and Inhibition. Cold Spring Harb. Perspect Biol..

[55] Jin X., Wang K., Liu H., Hu F., Zhao F., Liu J. (2016). Protection of Bovine Mammary Epithelial Cells from Hydrogen Peroxide-Induced Oxidative Cell Damage by Resveratrol. Oxid. Med. Cell Longev..

[56] Jakubczyk K., Dec K., Kałduńska J., Kawczuga D., Kochman J., Janda K. (2020). Reactive oxygen species-sources, functions, oxidative damage. Pol. Merkur. Lekarski..

[57] Weikard R., Kuehn C. (2018). Different mitochondrial DNA copy number in liver and mammary gland of lactating cows with divergent genetic background for milk production. Mol. Biol. Rep..

[58] Hadsell D.L., Torres D., George J., Capuco A.V., Ellis S.E., Fiorotto M.L. (2006). Changes in secretory cell turnover, and mitochondrial oxidative damage in the mouse mammary gland during a single prolonged lactation cycle suggest the possibility of accelerated cellular aging. Exp. Gerontol..

[59] Niu Y.J., Zhou W., Nie Z.W., Shin K.T., Cui X.S. (2020). Melatonin enhances mitochondrial biogenesis and protects against rotenone-induced mitochondrial deficiency in early porcine embryos. J. Pineal Res..

[60] Green D.R. (2021). Mitochondrial quality control: Just walk away. Cell Metab..

[61] Thoma A., Lyon M., Al-Shanti N., Nye G.A., Cooper R.G., Lightfoot A.P. (2020). Eukarion-134 Attenuates Endoplasmic Reticulum Stress-Induced Mitochondrial Dysfunction in Human Skeletal Muscle Cells. Antioxidants.

[62] Tanaka T., Nishimura A., Nishiyama K., Goto T., Numaga-Tomita T., Nishida M. (2020). Mitochondrial dynamics in exercise physiology. Pflug. Arch..

[63] Figge M.T., Reichert A.S., Meyer-Hermann M., Osiewacz H.D. (2012). Deceleration of fusion-fission cycles improves mitochondrial quality control during aging. PLoS Comput. Biol..

[64] Blanquer-Rosselló M.M., Santandreu F.M., Oliver J., Roca P., Valle A. (2015). Leptin Modulates Mitochondrial Function, Dynamics and Biogenesis in MCF-7 Cells. J. Cell Biochem..

[65] Peng K., Yang L., Wang J., Ye F., Dan G., Zhao Y., Cai Y., Cui Z., Ao L., Liu J. (2017). The Interaction of Mitochondrial Biogenesis and Fission/Fusion Mediated by PGC-1α Regulates Rotenone-Induced Dopaminergic Neurotoxicity. Mol. Neurobiol..

[66] Menon M.B., Dhamija S. (2018). Beclin 1 Phosphorylation-at the Center of Autophagy Regulation. Front. Cell Dev. Biol..

[67] Urbańska K., Orzechowski A. (2021). The Secrets of Alternative Autophagy. Cells.

[68] Wang Y., Zhang H. (2019). Regulation of Autophagy by mTOR Signaling Pathway. Adv. Exp. Med. Biol..

[69] Grumati P., Dikic I., Stolz A. (2018). ER-phagy at a glance. J. Cell Sci..

[70] Wang Y., Guo W., Xu H., Tang K., Zan L., Yang W. (2019). Melatonin suppresses milk fat synthesis by inhibiting the mTOR signaling pathway via the MT1 receptor in bovine mammary epithelial cells. J. Pineal Res..

[71] Zhang M., Chen D., Zhen Z., Ao J., Yuan X., Gao X. (2018). Annexin A2 positively regulates milk synthesis and proliferation of bovine mammary epithelial cells through the mTOR signaling pathway. J. Cell Physiol..

[72] Sun X., Chang R., Tang Y., Luo S., Jiang C., Jia H., Xu Q., Dong Z., Liang Y., Loor J.J. (2021). Transcription factor EB (TFEB)-mediated autophagy protects bovine mammary epithelial cells against H(2)O(2)-induced oxidative damage in vitro. J. Anim. Sci. Biotechnol..

[73] Audano M., Pedretti S., Caruso D., Crestani M., De Fabiani E., Mitro N. (2022). Regulatory mechanisms of the early phase of white adipocyte differentiation: An overview. Cell Mol. Life Sci..

[74] May K.S., den Hartigh L.J. (2021). Modulation of Adipocyte Metabolism by Microbial Short-Chain Fatty Acids. Nutrients.

[75] Lim S., Jang H.J., Park E.H., Kim J.K., Kim J.M., Kim E.K., Yea K., Kim Y.H., Lee-Kwon W., Ryu S.H. (2012). Wedelolactone inhibits adipogenesis through the ERK pathway in human adipose tissue-derived mesenchymal stem cells. J. Cell Biochem..

[76] Ando Y., Sato F., Fukunaga H., Iwasaki Y., Chiba Y., Tebakari M., Daigo Y., Kawashima J., Kamei J. (2019). Placental extract suppresses differentiation of 3T3-L1 preadipocytes to mature adipocytes via accelerated activation of p38 MAPK during the early phase of adipogenesis. Nutr. Metab..

[77] Yang T.T., Xiong Q., Enslen H., Davis R.J., Chow C.W. (2002). Phosphorylation of NFATc4 by p38 mitogen-activated protein kinases. Mol. Cell. Biol..

[78] Wang Y., Liu Y., Fan Z., Liu D., Wang F., Zhou Y. (2017). IGFBP2 enhances adipogenic differentiation potentials of mesenchymal stem cells from Wharton’s jelly of the umbilical cord via JNK and Akt signaling pathways. PLoS ONE.

[79] Zhang X., Cheng B., Jiang H., Liu C., Cao Z., Luan P., Wang N., Li H. (2021). Transcription Factor 21 Promotes Chicken Adipocyte Differentiation at Least in Part via Activating MAPK/JNK Signaling. Genes.

[80] Ackerman D., Tumanov S., Qiu B., Michalopoulou E., Spata M., Azzam A., Xie H., Simon M.C., Kamphorst J.J. (2018). Triglycerides Promote Lipid Homeostasis during Hypoxic Stress by Balancing Fatty Acid Saturation. Cell Rep..

[81] Wang W., Xin J., Yang X., Lam S.M., Shui G., Wang Y., Huang X. (2019). Lipid-gene regulatory network reveals coregulations of triacylglycerol with phosphatidylinositol/lysophosphatidylinositol and with hexosyl-ceramide. Biochim. Biophys. Acta Mol. Cell Biol. Lipids.

[82] Merrill A.H. (2002). De novo sphingolipid biosynthesis: A necessary, but dangerous, pathway. J. Biol. Chem..

[83] Sakamoto W., Canals D., Salamone S., Allopenna J., Clarke C.J., Snider J., Obeid L.M., Hannun Y.A. (2019). Probing compartment-specific sphingolipids with targeted bacterial sphingomyelinases and ceramidases. J. Lipid Res..

[84] Shiwani H.A., Elfaki M.Y., Memon D., Ali S., Aziz A., Egom E.E. (2021). Updates on sphingolipids: Spotlight on retinopathy. Biomed. Pharmacother..

[85] Zhao Y., Yu S., Zhao H., Li L., Li Y., Tu Y., Jiang L., Zhao G. (2022). Lipidomic profiling using GC and LC-MS/MS revealed the improved milk quality and lipid composition in dairy cows supplemented with citrus peel extract. Food Res. Int..

