# Supplementary material for: KRAS Affects the Lipid Composition by Regulating Mitochondrial Functions and MAPK Activation in Bovine Mammary Epithelial Cells
Source: Animals (Basel). 2022 Nov 8;12(22):3070. doi: 10.3390/ani12223070 (PMC9686882; doi:10.3390/ani12223070)
Supplement: Supplementary file 1 [file animals-12-03070-s001.zip › Supplementary Fig. S1, Table S1-S4, S7, Materials and Methoads.pdf]

animals

MDPI

Supplementary Materials

Supplementary Figure S-1

Figure S1. KRAS is expressed mainly in MAC-T cell nuclei S-1

Supplementary Tables S-2

Table S1. siRNA sequences used in this study S-2

Table S2. Primer sequences used in this study S-3

Table S3. Antibodies used for Western Blot and immunofluorescence (IF) S-5

Table S4. All values of folder changes (si-KRAS vs si-NC) S-7

Table S7. Relative folder changes of LC3B-II/LC3B-I S-9

Supplementary Materials and Methods S-10

1. Apoptosis detection S-10

2. RNA extraction and quantitative real-time reverse transcription polymerase chain reaction (qRT–PCR) S-10

3. Protein separation and Western blot analysis...S-10

4. Immunofluorescence...S-11

5. Triglyceride (TG) assay...S-11

6. Reactive oxygen species (ROS) assay...S-11

7. Mitochondrial morphology assay...S-11

8. ATP assay...S-12

9. Mitochondrial DNA (mtDNA) copy number assay.......................................S-12

10. Metabolomics..S-12

11. Lipidomics...S-14

animals

MDPI

Supplementary Figure

Figure S1. KRAS is expressed mainly in MAC-T cell nuclei

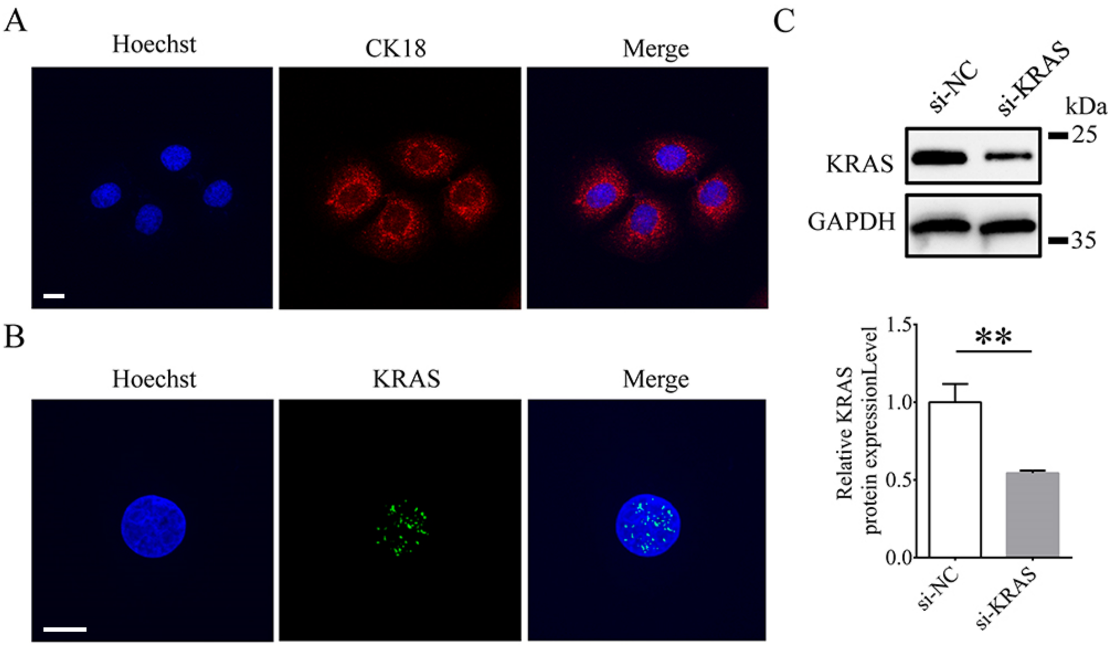

Figure S1. Identification of bovine mammary epithelial cells, intracellular localization of KRAS, and siRNA inhibition efficiency detection.

(**a**) Representative immunofluorescence images of cytokeratin 18 (CK18) in MAC-T cells. Bar = 10 µm. (**b**) Representative immunofluorescence images of KRAS in MAC-T cells. Bar = 10 µm. (**c**) Relative protein levels of KRAS in MAC-T cells with or without KRAS inhibition. Significant differences are represented with * ($P < 0.05$) and ** ($P < 0.01$).

animals

MDPI

Supplementary Table S1. siRNA sequences used in this study

siRNA name	Sequence (5' to 3') [a]
siRNA-KRAS	Sense-GCAAGAGUGCCUUGACGAUTT
siRNA-KRAS	Antisense-AUCGUCAAGGCACUCUUGCTT
siRNA-NC	Sense-UUCUCCGAACGUGUCACGUTT
siRNA-NC	Antisense-ACGUGACACGUUCGGAGAATT

[a] Additional "TT" was added to 3'-end region to enhance the silencing effect and the stability of siRNA.

Supplementary Table S2. Primer sequences used in this study

Genes	Accession number	Primer Sequence (5' to 3') [a]	Product length (bp)
β-ACTIN	NM_173979.3	F: CCCTGGAGAAGAGCTACGAG<br>R: GTAGTTTCGTGAATGCCGCAG	130
KRAS	NM_001110001.3	F: ACACAAAACAGGCTCAGGACT<br>R: GAAGGCATCGTCAACACCC	92
XDH	NM_173972.2	F: GGACAGAACCATCTCAGCCC<br>R: CGAGTCCTTGCCCAGTTTCT	214
CD36	NM_001278621.1	F: CAATCGAAACTGTGGGCTCATTG<br>R: TTAGAATCCCTCCAAACACAGCC	71
FABP3	NM_174313.2	F: TCGTCTTTCCCAACCTAGCC<br>R: TAGCAAAACCGACACCGAGT	118
ACSL1	XM_024986300.1	F: TGGCCCATATGTTTGAGAGA<br>R: GGGCCTTGAGATCATCCATA	108
ACSS2	NM_001105339.1	F: GGACTGAAACAGGGAAGCAA<br>R: CGCACAAGAGAAGCAACAAA	153
ACC	NM_174224.2	F: AGACAAACAGGGACCATT<br>R: AGGGACTGCCGAAACAT	141
FASN	NM_001012669.1	F: CCACGGCTGTCGGTAAT<br>R: CGCTCCCACTCATCCTG	163
SCD1	NM_173959.4	F: CTACACAACCACCACCACCA<br>R: CAGGGCACCCATCAGATAGT	301
GPAM	NM_001012282.1	F: ATTGACCCTTGGCACGATAG<br>R: AACAGCACCTTCCCACAAAG	188
LPIN1	NM_001206156.2	F: CCCTCCTGACATCCTGTGAA<br>R: AAACCTCCTCCTCGGTCCT	101
AGPAT1	NM_177518.1	F: AGTGTCATGTCTGAGGTCGC<br>R: CATGGAGCCGTTGTGGTTTC	93
COX5B	NM_001034046.2	F: TCCATCACCAACAAGCGGAT<br>R: TGCAGCCAGAACCAGATGAC	74

NDUFS8	NM_001302669.1	F: ACCAAGTGCATCTACTGCGG<br>R: CGGTAAAGGTAGTCGGCCTG	188
SDHB	NM_001040483.1	F: TGGTCGCGCTCTCCTTGA<br>R: AGCATCCAGCACCATAGGAC	215
ATP5F1A	NM_174684.2	F: TGGTGATGGTATTGCTCGGG<br>R: GGCATCAACTACACGACCCA	229
UQCRB	NM_001034797.2	F: GCCGGCCAGCTGTTTCA<br>R: AACCCGGCAGCGTTGTAATA	73
NRF1	NM_001098002.2	F: GAGGTGGAACAAAACTGGGC<br>R: CTCTTTTATTGCCCACCCCTG	189
POLG	XM_024982309.1	F: CCAGTGCCTCAGGAAGGAAG<br>R: CGTATCGCCTTTCCATCCCA	74
TFAM	NM_001034016.2	F: CAGGAAGCTAGGGATGGCAC<br>R: AGCTTCCGGTATTGAGACCT	300
TFB1M	NM_001076896.2	F: TGCCCAAACCAGAGAATGTT<br>R: GTAGGTCGGAGAGTCGGGT	98
BECN1	NM_001033627.2	F: GCCTCTGAAACTGGACACGA<br>R: GGACATCATCCTGGCTGGG	199
ATG7	NM_001083795.2	F: GCACGGCTTGTTCTTCCAAAG<br>R: TTGTTTATACACCTCAGCAGCC	155

[a] F: Forward primer; R: Reverse primer.

animals

MDPI

Supplementary Table S3. Antibodies used for Western Blot and immunofluorescence (IF)

Primary antibodies		
**Antibody**	**Company[a]**	**Cat. Number**
KRAS	ThermoFisher Scientific	415700
LC3B	Abcam	ab192890
mTOR	Abcam	ab2732
p-mTOR	Cell Signaling Technology	2971S
EGF	Affinity Biosciences	DF2225
EGFR	Abcam	ab52894
IGF1R	Affinity Biosciences	AF6124
TGFβR1	Abcam	ab31013
FABP4	Abcam	ab92501
SREBP1	Abcam	ab28481
PPARG	Cell Signaling Technology	2443S
GRP78	Wanleibio	WL03157
ATF6	Affinity Biosciences	DF6009
CHOP	Cell Signaling Technology	5554S
ERK	Abcam	ab17942
p-ERK	Cell Signaling Technology	4370S
JNK	Abcam	ab179461
p-JNK	Abcam	ab124956
p38	Abcam	ab31828
p-p38	Cell Signaling Technology	4511S
CALNEXIN	Abcam	ab112995
CASPASE 3	Proteintech	19677-1-AP
cleaved-CASPASE 3	CST	9664S
MFN1	Proteintech	13798-1-AP
MFN2	Proteintech	12186-1-AP
OPA1	Proteintech	27733-1-AP
DRP1	CST	8570S
β-ACTIN	Abcam	4970S
α-TUBULIN	Abcam	ab52866
GAPDH	Cell Signaling Technology	2118S
**Secondary antibodies**		
**Antibody**	**Company[a]**	**Cat. Number**
Goat anti-rabbit IgG	Bioworld Technology Inc.	BS13278
Goat anti-mouse IgG	Bioworld Technology Inc.	BS12478
Anti-rabbit IgG (Alexa Fluor 488)	Cell Signaling Technology	4412S
Anti-Mouse IgG (Alexa Fluor 555)	Abcam	ab150118

Anti-rabbit IgG (Alexa Fluor 647)	Abcam	ab150075

[a] ThermoFisher Scientific, Waltham, MA, USA.
Cell Signaling Technology, Beverly, MA, USA.
Abcam, Cambridge, MA, USA
Bioworld Technology Inc., Louis Park, MN, USA.
Affinity Biosciences, Changzhou, China.
Wanleibio, Shenyang, China.
Proteintech, Wuhan, China.
[b] Anti-rabbit IgG (H+L)-Alexa Fluor® 488 Conjugate.

Supplementary Table S4. All values of folder changes (si-KRAS vs si-NC)

**Figures**	**Indexes**	**Folder changes (si-KRAS vs si-NC)**	***P* value**
Figure. S1C	*KRAS* mRNA	0.54 ± 0.01	$P < 0.01$
Figure. 3B	Lipid droplets	1.50 ± 0.61	$P < 0.01$
Figure. 3C	TG level	1.53 ± 0.10	$P < 0.01$
Figure. 3E	EGF protein	1.44 ± 0.17	$P = 0.019$
	EGFR protein	1.16 ± 0.10	$P = 0.012$
	IGF1R protein	1.19 ± 0.10	$P = 0.037$
	TGFBR1 protein	0.90 ± 0.01	$P < 0.01$
Figure. 3F	FABP4 protein	1.53 ± 0.23	$P = 0.042$
	PPARG protein	1.22 ± 0.12	$P = 0.033$
	SREBP1 protein	1.14 ± 0.02	$P < 0.01$
Figure. 3G	*XDH* mRNA	1.37 ± 0.11	$P < 0.01$
	CD36 mRNA	1.40 ± 0.14	$P = 0.012$
	FABP3 mRNA	1.79 ± 0.13	$P < 0.01$
	ACSL1 mRNA	1.17 ± 0.09	$P = 0.039$
	ACSS2 mRNA	1.53 ± 0.25	$P = 0.027$
	ACC mRNA	1.54 ± 0.12	$P < 0.01$
	FASN mRNA	1.23 ± 0.03	$P = 0.015$
	SCD1 mRNA	1.21 ± 0.07	$P < 0.01$
	GPAM mRNA	1.50 ± 0.18	$P = 0.027$
	LPIN1 mRNA	1.43 ± 0.17	$P = 0.012$
	AGPAT1 mRNA	1.24 ± 0.11	$P = 0.028$
Figure. 4A	mtDNA level	1.58 ± 0.88	$P < 0.01$
Figure. 4B	ATP level	1.53 ± 0.10	$P < 0.01$
Figure. 4C	*COX5B* mRNA	1.38 ± 0.14	$P < 0.01$
	NDUFS8 mRNA	1.51 ± 0.17	$P < 0.01$
	SDHB mRNA	1.47 ± 0.24	$P = 0.031$
	ATP5F1A mRNA	1.50 ± 0.24	$P = 0.021$
	UQCRB mRNA	1.23 ± 0.10	$P = 0.022$
	NRF1 mRNA	1.49 ± 0.16	$P < 0.01$
	POLG mRNA	3.35 ± 0.37	$P < 0.039$
	TFAM mRNA	1.65 ± 0.08	$P < 0.01$
	TFB1M mRNA	1.40 ± 0.10	$P < 0.01$
Figure. 4F	MFN1	1.09 ± 0.02	$P < 0.01$
	MFN2	2.19 ± 0.29	$P < 0.01$
	OPA1	1.24 ± 0.05	$P < 0.01$
	DRP1	1.32 ± 0.04	$P < 0.01$
Figure. 5A	ROS level	1.19 ± 0.10	$P = 0.031$
Figure. 5C	GRP78 protein	1.34 ± 0.12	$P < 0.01$
	CHOP protein	1.36 ± 0.09	$P < 0.01$

	ATF6 protein	1.08 ± 0.06	*P* = 0.286
Figure. 5D	CAT level	1.38 ± 0.11	*P* < 0.01
	GSH level	1.81 ± 0.10	*P* < 0.01
	T-AOC level	1.09 ± 0.02	*P* < 0.01
Figure. 5F	Apoptosis level	1.22 ± 0.21	*P* = 0.161
Figure. 5H	CASPASE 3	1.05 ± 0.05	*P* = 0.261
	cleaved-CASPASE 3	0.85 ± 0.13	*P* = 0.119
Figure. 6D	p-mTOR/mTOR level	0.79 ± 0.03	*P* < 0.01
Figure. 6E	*BECN 1* mRNA	1.32 ± 0.17	*P* = 0.040
	ATG7 mRNA	1.26 ± 0.11	*P* = 0.021
Figure. 7	p-ERK/ERK level	0.80 ± 0.06	*P* < 0.01
	p-JNK/JNK level	1.15 ± 0.03	*P* < 0.01
	p-p38/p38 level	0.89 ± 0.05	*P* = 0.032

Supplementary Table S7. Relative folder changes of LC3B-II/LC3B-I

	si-NC	si-KRAS	Chloroquine
Relative folder changes	1.00 ± 0.03	1.25 ± 0.05	1.82 ± 0.10
P valve (vs si-NC)		*P* = 0.012	*P* < 0.01

animals

MDPI

Supplementary Materials and Methods

1. Apoptosis detection

MAC-T cells were plated in 6-well plates. 24 h after cell transfection, the cells were washed with PBS and digested by trypsin for collection with centrifuge tube. Then, 100 μL of PBS were added to each centrifuge tube, followed by addition of 5 μL of FITC solution and 5 μL of propidium iodide. The cells were incubated for 10 min in the dark at room temperature. The cell samples were analyzed using the flow cytometry (Beckman Coulter, Brea, CA, USA).

2. RNA extraction and quantitative real-time reverse transcription polymerase chain reaction (qRT–PCR)

MAC-T cells were washed once in 1× phosphate-buffered saline (PBS), and total RNA was extracted using TRIzol Reagent (Life Technologies, Carlsbad, CA, USA). First-strand cDNA was synthesized from 1 μg of total RNA by using a fast reverse transcription kit (TIANGEN, Beijing, China) according to the manufacturer's instructions. Each 20 μL qRT–PCR mixed system included 8 μL of deionized water, 10 μL of 2× SuperReal PreMix Plus (TIANGEN), 1 μL of cDNA, and 0.5 μL each of forward and reverse primers (10 μM). The qRT–PCR conditions included denaturation at 95 °C for 180 s and 40 cycles of 95 °C for 10 s, 60 °C for 20 s, and 72 °C for 30 s. Gene expression was quantified using the Mastercycler ep realplex (Eppendorf, Hamburg, Germany) and $2^{-\triangle\triangle CT}$ method with *β-ACTIN* as the standard.

3. Protein separation and Western blot analysis

MAC-T cells were trypsinized, washed once in medium and once in PBS, and were pelleted for 5 min at 500× *g*. Then, RIPA buffer (Solarbio, Beijing, China) containing 1% PMSF (100 mM, Solarbio) was added to the cell suspension and mixed well. After that, the cells were ultrasonicated for 60 s (3 times per s). Next, the sample was lysed on ice for 30 min. After lysis, the mixture was centrifuged for 10 min at 16,000× *g* to collect the protein-containing supernatant. The samples and SDS–PAGE loading buffer (4×, with β-mercaptoethanol; Solarbio) were mixed and incubated at 95 °C for 5 min. The protein samples were separated by SDS–PAGE and then transferred to a polyvinylidene fluoride membrane (0.45 μm, Millipore, Bedford, MA, USA). Next, the membranes were transferred to 5% BSA blocking solution at room temperature, blocked for 2 h, and incubated with the corresponding primary antibodies, which were diluted to a suitable concentration with blocking solution at 4 °C overnight. After the membranes were washed 3 times with 1× Tris-buffered saline that contained 0.5% Tween-20 for 10 min each, the membranes were incubated at room temperature for 1 h with the corresponding secondary antibodies. Finally, a Tanon 5200 image analyzer (Tanon, Shanghai, China) and ImageJ software (NIH, Bethesda, MD, USA) were used for image capture and gray value analysis. The GAPDH loading controls were performed on the same membrane unless indicated otherwise.

4. Immunofluorescence

Briefly, cell culture slides (Nest Biotechnology) were added to 12-well plates before cell seeding. Then, a total of 1 × 10^5 cells were seeded on the slides with cell culture medium. After transfection, the cells were fixed in PBS with 4% paraformaldehyde for 30 min and were permeabilized with PBS with 0.3% Triton X-100 at room temperature for 30 min. Then, the cells were blocked in PBS with 5% BSA at room temperature for 2 h. Next, the cells were incubated with the indicated primary antibodies overnight at 4 °C. After washing three times with PBS, the cells were incubated at 37 °C for 1 h with related secondary antibodies. The cells were then stained with 10 μg/mL Hoechst 33342 for 10 min and were washed three times with PBS. Finally, the cell culture slides were mounted onto the slides and examined under a confocal laser scanning microscope (Carl Zeiss, Jena, Germany).

5. Triglyceride (TG) assay

All experiments were performed according to the manufacturer's instructions. The amount of intracellular TG relative to the total protein was detected using a tissue/cell triacylglycerol assay kit (Applygen Technologies, Beijing, China) at 550 nm and a BCA protein assay kit (Beyotime) at 562 nm with a microplate reader (BioTek Instruments), respectively, according to the manufacturer's instructions. The relative TG level was calculated as optical density $(OD)_{TG\text{-}550}$ values relative to the total protein level.

6. Reactive oxygen species (ROS) assay

24 h after cell transfection, the MAC-T cells were washed with PBS and digested by trypsin for collection. After collection, the cells were incubated (and rotated upside down every 5 min) in PBS containing 10 μM 2',7'-dichlorofluorescein diacetate (DCFH-DA, Beyotime) for 30 min at 37 °C in a 5% CO_2 atmosphere. Then, the cells were collected by centrifugation and washed with PBS to remove DCFH-DA that did not enter the cells. The samples were then placed on ice and analyzed by flow cytometry within 1 h of treatment.

7. Mitochondrial morphology assay

Briefly, cell culture slides (Nest Biotechnology) were added to 12-well plates before cell seeding. After transfection, MAC-T cells were incubated in PBS that contained 2 μg/mL Bodipy (Invitrogen) and 200 nM MitoTracker Red CMXRos (Invitrogen) for 30 min at 37 °C in an atmosphere of 5% CO_2. Then, the cells were fixed in PBS with 4% paraformaldehyde for 30 min. After washing three times with PBS, the cells were stained with 10 μg/mL Hoechst 33342 for 10 min and washed three times with PBS. Finally, the cell culture slides were mounted onto the slides and examined under a confocal laser scanning microscope (Carl Zeiss, Jena, Germany).

8. ATP assay

According to the manufacturer's instructions, intracellular ATP levels were measured using an enhancing ATP detection kit (Beyotime). Briefly, after transfection, MAC-T

cells were lysed with 200 μL of lysis buffer. Next, the cell lysates were centrifuged at 12,000 rpm at 4 °C for 5 min, and the supernatant was obtained for subsequent analysis. Then, 100 μL of ATP working solution and 20 μL of supernatant were added to 96-well plates and were quantified by obtaining measurements with a microplate reader (BioTek Instruments).

9. Mitochondrial DNA (mtDNA) copy number assay

According to the manufacturer's requirements, the whole genome was extracted by using the QIAamp Fast DNA Tissue Kit (Qiagen, Hilden, Germany). qRT–PCR was performed by using SuperReal PreMix Plus (TIANGEN). Amplification was conducted as follows: 95 °C for 3 min, followed by 40 cycles of 95 °C for 10 s, 60 °C for 20 s, and 72 °C for 30 s. The relative quantification method was used to calculate the mtDNA copy number. Forward primer: GAACCACTACGACCCGCTAC. Reverse primer: ACGGCTAGGCTTGATATGGC.

10. Metabolomics

10.1 Metabolite Extraction

The samples were accurately weighed, and the metabolites extracted using a 400 μL methanol:water (4:1, v/v) solution. The mixture was allowed to settle at -10 °C and treated by High throughput tissue crusher Wonbio-96c (Shanghai wanbo biotechnology co., LTD) at 50 Hz for 6 min, then followed by vortex for 30 s and ultrasound at 40 kHz for 30 min at 5 °C. The samples were placed at -20 °C for 30 min to precipitation. After centrifugation at 13000× *g* at 4 °C for 15min, the supernatants were carefully transferred to sample vials for LC-MS/MS analysis.

10.2 Quality control sample

As a part of the system conditioning and quality control process, a pooled quality control sample (QC) was prepared by mixing equal volumes of all samples. The QC samples were disposed and tested in the same manner as the analytic samples. It helped to represent the whole sample set, which would be injected at regular intervals in order to monitor the stability of the analysis.

10.3 UHPLC-MS/MS analysis.

Chromatographic separation of the metabolites was performed on a Thermo UHPLC system equipped with an ACQUITY UPLC HSS T3 (100 mm × 2.1 mm i.d., 1.8 μm; Waters Corporation, Milford, MA, USA).

The mobile phases consisted of 0.1% formic acid in 95% acetonitrile solution (solvent A) and 0.1% formic acid in acetonitrile:isopropanol (1:1, v/v; solvent B). For equilibrating the systems, the solvent gradient changed according to the following conditions: from 0 to 3.5 min, 100% (A): 0% (B) to 75.5% (A): 24.5% (B); from 3.5 to 5 min, 75.5% (A): 24.5% (B) to 35% (A): 65% (B); from 5 to 5.5 min, 35% (A): 65% (B) to 0% (A): 100% (B); from 5.5 to 7.4 min, keep unchanged; from 7.4 to 7.6 min, 0% (A): 100% (B) to 48.5% (A): 51.5% (B); from 7.6 to 7.8 min, 48.5% (A): 51.5% (B) to 100% (A): 0% (B); from 7.8 to 10 min, 100% (A): keep unchanged. The flow

rate changed according to the following conditions: from 0 to 5.5 min, the flow rate was set to 0.4 mL/min; from 5.5 to 7.4 min, 0.4 mL/min to 0.6 mL/min; from 7.4 to 7.6 min, keep unchanged; from 7.6 to 7.8 min, 0.6 mL/min to 0.5 mL/min; from 7.8 to 9 min, 0.5 mL/min to 0.4 mL/min; from 9 to 10 min, keep unchanged. The sample injection volume was 2 μL. The column temperature was maintained at 40 °C. During the period of analysis, all these samples were stored at 4 °C.

The mass spectrometric data was collected using a Thermo UHPLC-Q Exactive Mass Spectrometer equipped with an electrospray ionization (ESI) source operating in either positive or negative ion mode. The optimal conditions were set as followed: Aus gas heater temperature, 425 °C; Sheath gas flow rate 50 arb; Aus gas flow rate 13 arb; ion-spray voltage floating (ISVF): -3500V in negative mode and 3500V in positive mode, respectively; Normalized collision energy, 20-40-60V rolling for MS/MS. The detection was carried out over a mass range of 70-1050 m/z.

10.4 Data preprocessing and annotation

After UHPLC-MS/MS analyses, the raw data were imported into the ProgenesisQI (WatersCorporation, Milford, USA) for peak detection and alignment. The preprocessing results generated a data matrix that consisted of the retention time (RT), mass-to-charge ratio (m/z) values, and peak intensity. After filtering, minimum metabolite values were imputed for specific samples in which the metabolite levels fell below the lower limit of quantitation and each metabolic features were normalized by sum. The internal standard was used for data QC (reproducibility), Metabolic features which the relative standard deviation (RSD) of QC > 30% were discarded. Following normalization procedures and imputation, statistical analysis was performed on log transformed data to identify significant differences in metabolite levels between comparable groups. Mass spectra of these metabolic features were identified by using the accurate mass, MS/MS fragments spectra and isotope ratio difference with searching in reliable biochemical databases as Human metabolome database (HMDB) (http://www.hmdb.ca/) and Metlin database (https://metlin.scripps.edu/). Concretely, the mass tolerance between the measured m/z values and the exact mass of the components of interest was ±10 ppm.

10.5 Multivariate statistical analysis

A multivariate statistical analysis was performed using ropls (Version1.6.2, http://bioconductor.org/packages/release/bioc/html/ropls.html) R package from Bioconductor on Majorbio Cloud Platform (https://cloud.majorbio.com). Orthogonal partial least squares discriminate analysis (OPLS-DA) was used for statistical analysis to determine global metabolic changes between comparable groups. All of the metabolite variables were scaled to pareto Scaling prior to conducting the OPLS-DA. The model validity was evaluated from model parameters R2 and Q2, which provide information for the interpretability and predictability, respectively, of the model and avoid the risk of over-fitting. Variable importance in the projection (VIP) were calculated in OPLS-DA model. *P* values were estimated with paired Student's t-test on Single dimensional statistical analysis.

10.6 Differential metabolites analysis

Statistically significant among groups were selected with *P* value < 0.05, log_2(FC) > 1, and VIP > 1. Differential metabolites among two groups were summarized, and mapped into their biochemical pathways through metabolic enrichment and pathway analysis based on database search (KEGG, http://www. genome.jp/kegg/). These metabolites can be classified according to the pathways they involved or the functions they performed. Enrichment analysis was usually to analyze a group of metabolites in a function node whether appears or not. The principle was that the annotation analysis of a single metabolite develops into an annotation analysis of a group of metabolites. Scipy.stats (Python packages, https://docs.scipy.org/doc/scipy/) was exploited to **identify statistically significantly enriched pathway using Fisher's exact test.**

11. Lipidomics

11.1 Lipid Extraction

The samples were accurately weighed, and the metabolites extracted using a 280 μL methanol:water (2:5) solution and 400 μL methyl tert-butyl ether. The mixture was allowed to settle at -10 °C and treated by High throughput tissue crusher Wonbio-96c (Shanghai wanbo biotechnology co., LTD) at 50 Hz for 6 min, then ultrasound at 40 kHz for 30 min at 5 °C. The samples were placed at -20 °C for 30 min to precipitation. After centrifugation at 13000× *g* at 4 °C for 15min, 350 μL of supernatants were transferred to EP tube and blow-dried with nitrogen. Then, 100 μL methanol:water (1:1) solution was added to EP tube for redissolving, then followed by vortex for 30 s and ultrasound at 40 kHz for 5 min at 5 °C. After centrifugation at 13000× g at 4 °C for 10 min, the supernatants were carefully transferred to sample vials for LC-MS/MS analysis.

11.2 Quality control sample

As a part of the system conditioning and quality control process, a pooled quality control sample (QC) was prepared by mixing equal volumes of all samples. The QC samples were disposed and tested in the same manner as the analytic samples. It helped to represent the whole sample set, which would be injected at regular intervals in order to monitor the stability of the analysis.

11.3 UHPLC-MS/MS analysis.

Chromatographic separation of the metabolites was performed on a Thermo UHPLC system equipped with an Accucore C30 column (100 mm × 2.1 mm i.d., 2.6 μm; Thermo).

The mobile phases consisted of 0.1% formic acid and 10 m*M* ethanolamine in 50% acetonitrile solution (solvent A), and 0.02% formic acid and 2 m*M* ethanolamine in acetonitrile:isopropanol:water (10/88/2; solvent B). For equilibrating the systems, the solvent gradient changed according to the following conditions: from 0 to 4 min, 65% (A): 35% (B) to 40% (A): 60% (B); from 4 to 12 min, 40% (A): 60% (B) to 15% (A): 85% (B); from 12 to 15 min, 15% (A): 85% (B) to 0% (A): 100% (B); from 15 to 17 min, keep unchanged; from 17 to 18 min, 0% (A): 100% (B) to 65% (A): 35% (B);

from 18 to 20 min, keep unchanged. The sample injection volume was 2 μL and the flow rate was set to 0.4 mL/min. The column temperature was maintained at 40ºC. During the period of analysis, all these samples were stored at 4ºC.
The mass spectrometric data was collected using a Thermo UHPLC-Q Exactive Mass Spectrometer equipped with an ESI source operating in either positive or negative ion mode. The optimal conditions were set as followed: Aus gas heater temperature, 370 °C; Sheath gas flow rate 60 psi; Aus gas flow rate 20 psi; ISVF: -3000V in negative mode and 3000V in positive mode, respectively; Normalized collision energy, 20-40-60V rolling for MS/MS.

11.4 Data preprocessing and annotation
After UHPLC-MS/MS analyses, the raw data were imported into the Lipidsearch (Thermo) for peak detection and alignment. The preprocessing results generated a data matrix that consisted of the RT, m/z values, and peak intensity. After filtering, minimum metabolite values were imputed for specific samples in which the metabolite levels fell below the lower limit of quantitation and each metabolic features were normalized by sum. The internal standard was used for data QC (reproducibility), Metabolic features which the RSD of QC > 30% were discarded. Following normalization procedures and imputation, statistical analysis was performed on log transformed data to identify significant differences in metabolite levels between comparable groups. Mass spectra of these metabolic features were identified by using the accurate mass, MS/MS fragments spectra and isotope ratio difference with searching in reliable biochemical databases as HMDB (http://www.hmdb.ca/) and Metlin database (https://metlin.scripps.edu/). Concretely, the mass tolerance between the measured m/z values and the exact mass of the components of interest was ±10 ppm.

11.5 Multivariate statistical analysis
A multivariate statistical analysis was performed using ropls (Version1.6.2, http://bioconductor.org/packages/release/bioc/html/ropls.html) R package from Bioconductor on Majorbio Cloud Platform (https://cloud.majorbio.com). OPLS-DA was used for statistical analysis to determine global metabolic changes between comparable groups. All of the metabolite variables were scaled to pareto Scaling prior to conducting the OPLS-DA. The model validity was evaluated from model parameters R2 and Q2, which provide information for the interpretability and predictability, respectively, of the model and avoid the risk of over-fitting. VIP were calculated in OPLS-DA model. *P* values were estimated with paired Student's t-test on Single dimensional statistical analysis.

11.6 Differential lipid metabolites analysis
Statistically significant among groups were selected with P value < 0.05, $\log_2(FC) > 1$, and VIP > 1. Differential metabolites among two groups were summarized, and mapped into their biochemical pathways through metabolic enrichment and pathway analysis based on database search (KEGG, http://www. genome.jp/kegg/). These metabolites can be classified according to the pathways they involved or the functions they

animals

MDPI

performed. Enrichment analysis was usually to analyze a group of metabolites in a function node whether appears or not. The principle was that the annotation analysis of a single metabolite develops into an annotation analysis of a group of metabolites. Scipy.stats (Python packages, https://docs.scipy.org/doc/scipy/) was exploited to identify statistically significantly enriched pathway using Fisher's exact test.
